# Volumetric and Surface Properties of Short Chain Alcohols in Aqueous Solution–Air Systems at 293 K

**DOI:** 10.1007/s10953-012-9935-z

**Published:** 2012-12-04

**Authors:** Aleksandra Chodzińska, Anna Zdziennicka, Bronisław Jańczuk

**Affiliations:** Department of Interfacial Phenomena, Faculty of Chemistry, Maria Curie-Skłodowska University, Maria Curie-Skłodowska Sq. 3, 20-031 Lublin, Poland

**Keywords:** Alcohols, Adsorption, Alcohol molar volume

## Abstract

From measurements of the surface tension, density, viscosity and light scattering of aqueous solutions of methanol, ethanol and propanol at 293 K, their activity in the surface monolayer, surface excess concentration, and apparent and partial molar volume were determined. The surface excess concentration of alcohols at the water–air interface was determined from the Gibbs equation by using both the alcohol's activity and their molar fraction in the bulk phase and recalculated by using the Guggenheim–Adam equation. The values of the surface excess concentration determined from the Gibbs equation were also applied to determine the standard Gibbs energy of alcohol adsorption at the water–air interface from Langmuir’s equation and compared to those determined from that of Aronson and Rosen.

**Electronic supplementary material:**

The online version of this article (doi:10.1007/s10953-012-9935-z) contains supplementary material, which is available to authorized users.

## Introduction

Short chain alcohols are applied as solvents, co-solvents or co-surfactants and, among other things, they can change volumetric and surface properties of surfactants [1–8]. Alcohol–water mixtures have always been seen as interesting due to their anomalous behavior such as the existence of a viscosity–composition maximum and decrease of the partial molar volume in comparison to their volume in the “pure” alcohol state. This behavior depends on the solution microstructure.

The microstructure of the bulk phase of aqueous solutions of alcohols is governed by the hydrogen bond, hydrophobic interaction and hydration, which are reflected in the enthalpy and entropy contributions in the free enthalpy of solution [9]. For a dilute solution, hydrophobic hydration, consisting of water structure enhancement accompanied by entropy decrease, occurs mainly in any mixture of alcohol and water. On the other hand, when the amount of alcohol molecules is greater various structures appear, depending on the alkyl group's shape and alcohol concentration.

From fluorescence probe investigations Zana and Eljebari [10] stated that alcohol self-association takes place in aqueous solution. The resulting alcohol aggregates appear to be short-lived and to have some properties of classical surfactant micelles. However, the concentration at which alcohols aggregation takes place, determined by other investigators [11–13], differs somewhat from that of Zana and Eljebari [10].

Yoshida and Yamaguchi [14] using low-frequency Raman spectroscopy proved that the structure of aqueous solutions of short chain alcohols is characterized by individual alcohol aggregates and water clusters without a significant amount of alcohol–water mixed aggregates.

Roney et al. [15], based on low-frequency Raman, small-angle X-ray scattering and small-angle neutron scattering studies of various concentrations of aqueous *n*-propanol at room temperature, also stated that both water and *n*-propanol form single-component aggregates in solution.

However, in contrast to these conclusions, Alavi et al. [16] suggested that the small alcohols ethanol, 1-propanol, and 2-propanol form strong hydrogen bonds with water molecules, and are usually known as inhibitors for clathrate hydrate formation. Fidler and Rodger [17] stated that even methanol molecules aggregate in water, which was proved by the thermodynamic arguments of Tamaka and Gubbins [18].

Independently of the opinions about the structure of the aqueous solutions of short chain alcohols, it is known that their structure influences the volumetric properties of solutions, which is reflected, among other things, by the density and viscosity values. Using the density data it is possible to calculate the apparent and partial molar volumes of both water and the alcohols, and deviations from ideality can be observed. In the literature it is possible to find many data for partial and excess volume changes as a function of composition of aqueous solutions of short chain alcohols [19–23]. Many authors stated that there is a minimum in the alcohol partial excess volume [19–21]. However, there are some different opinions about the value of this minimum for the particular alcohols and at which alcohol concentration it appears [19–21]. This minimum should be connected with the structures of water and alcohol molecules, but it is difficult to find an univocal explanation of this phenomenon.

It is known that there is a correlation between the structure of aqueous solution of a surface active agent and its tendency to adsorb at the water–air interface [1, 24, 25].

Among other things, Yano [25] stated that the maximum of the surface excess concentration of short chain alcohols does not depend on their kind and exactly coincides with the alcohol molar fraction in the bulk phase corresponding to the minimal values of their excess partial molar volumes. In contrast to Yano [25], Lavi and Marmur [26] suggested that two maxima may be expected on the adsorption isotherm of alcohols.

The surface excess concentration of alcohol at water–air interface is commonly determined from the Gibbs adsorption equation. To evaluate this concentration quantitatively a reliable data set of two parameters, the surface tension and the activity of alcohol in the bulk phase should be known. In most previous works the surface excess of alcohol concentration was calculated using the molar concentration instead of the alcohol's activity. Strey et al. [27], Lavi and Marmur [26] and Yano [25] took into account the alcohol activity in the bulk phase in their calculations of alcohol excess concentration from the Gibbs equation. However, they pointed out clearly that the activity of alcohols determined from the Laar equation [28], on the basis of their partial pressure over the solution, does not give direct information about the ideality of aqueous solutions of alcohols. As is known, two activity sets were defined [28], one called symmetric and the other asymmetric. The former is based on the assumption that the solvent and solute activity coefficients go to unity as their molar fractions approach unity. The definition of the solvent activity coefficient in the case of the latter is the same, but for solute it is assumed that its activity coefficient goes to unity if the molar fraction is close to zero. It should be taken into account if the relationship between the activity of a given solute is considered in the bulk phase and surface region [29]. In the literature there is lack of a clear explanation of this problem not only with regard to calculations of the surface excess concentration from the Gibbs equation, but also in consequence calculations of thermodynamic functions of the adsorption process. Taking into account the fact that in the literature there is no agreement concerning the volume and surface properties of short chain alcohols, the main purpose of our paper is to discuss this problem basing on measurements of the surface tension, density, viscosity and light scattering of aqueous solutions of these short chain alcohols.

### Gibbs Isotherm of Adsorption

For muliticomponet systems, including two phases in isobaric and isothermal processes, according to the Gibbs theory, the following equation is fulfilled for the surface region [29]:

1 $$ dG^{S} = - S^{S} dT + V^{S} dp + \gamma dA + \sum\limits_{i} {\mu_{i} dn_{i}^{S} } $$

where *G* ^*S*^ is the Gibbs energy of the surface region, *S* ^S^ is the entropy of the surface region, *V* ^*S*^ is the volume of the surface region, *γ* is the surface or interface tension, *A* is the interfacial area, *μ*_*i*_ is the chemical potential of the *i* component in the surface region and $n_{i}^{s}$ is the number moles of component *i*.

For constant *T* and *p*, in the equilibrium state of the reversible adsorption process, from Eq. 1 it results that:

2 $$ d\gamma = - \sum\limits_{i} {\Upgamma_{i} d\mu_{i} } $$

where $\Upgamma_{i} = n_{i}^{S} /A$ is the surface excess concentration of component *i* in the surface region. Equation 2 is the general Gibbs isotherm of adsorption.

In the literature it is possible to find improper applications of this equation for determination of surface excess concentration of some surface active agents at the water–air or water–oil interface. It is particularly evident in the case of solutions in which the components are mixing in their total concentration range, for example, aqueous solutions of short chain alcohols.

It is known that the standard chemical potential can be defined in two different ways. Thus, for the surface region *S* and bulk *B* phase the chemical potential can be expressed by the following equations, respectively [30]:

3a $$ \mu_{i}^{S} = \mu_{i}^{S(0)} + RT\ln a_{i}^{S} - \gamma_{i} \omega_{i} $$

3b $$ \mu_{i}^{S} = \mu_{i}^{S(0)} + RT\ln x_{i}^{S} f_{i}^{S} - \gamma_{i} \omega_{i} $$

or

4a $$ \mu_{i}^{S} = \mu_{i}^{S(\Uptheta )} + RT\ln a_{i}^{*S} - \gamma_{i} \omega_{i} $$

4b $$ \mu_{i}^{S} = \mu_{i}^{S(\Uptheta )} + RT\ln x_{i}^{S} f_{i}^{*S} - \gamma_{i} \omega_{i} $$

and

5a $$ \mu_{i}^{B} = \mu_{i}^{B(0)} + RT\ln a_{i}^{B} $$

5b $$ \mu_{i}^{B} = \mu_{i}^{B(0)} + RT\ln x_{i}^{B} f_{i}^{B} $$

or

6a $$ \mu_{i}^{B} = \mu_{i}^{B(\Uptheta )} + RT\ln a_{i}^{*B} $$

6b $$ \mu_{i}^{B} = \mu_{i}^{B(\Uptheta )} + RT\ln x_{i}^{S} f_{i}^{*B} $$

where *T* is temperature, *R* the gas constant, *a* the activity, *f* the activity coefficient and $\mu_{i}^{\text{(O)}}$ the standard chemical potential if $f_{i} \to 1$ for $x_{i} \to 1$, $\mu_{i}^{(\Uptheta )}$ is the standard chemical potential if $f_{i}^{*} \to 1$ for $x_{i} \to 0$, $\gamma_{i}$ is the surface tension of pure component *i* and $\omega_{i}$ is the molar surface area of component *i*.

Because in the equilibrium state $\mu_{i}^{B} = \mu_{i}^{S}$, by differentiating Eqs. 5a and 6a we obtain the Gibbs isotherm equation in the forms:

7a $$ \Upgamma_{i} = - \frac{{a_{i} }}{RT}\left( {\frac{\partial \gamma }{{\partial a_{i} }}} \right)_{{T,p,n_{j \ne i} }} $$

and

7b $$ \Upgamma_{i} = - \frac{{a_{i}^{*} }}{RT}\left( {\frac{\partial \gamma }{{\partial a_{i}^{*} }}} \right)_{{T,p,n_{j \ne i} }} $$

From Eqs. 7a and 7b:

8 $$ \frac{{a_{i}^{*} }}{RT}\left( {\frac{\partial \gamma }{{\partial a_{i}^{*} }}} \right)_{{T,p,n_{j} \ne i}} = \frac{{a_{i} }}{RT}\left( {\frac{\partial \gamma }{{\partial a_{i} }}} \right)_{{T,p,n_{j \ne i} }} $$

For dilute solutions for which $f_{i}^{*} \approx 1$ Eq. 8 gives:

9 $$ \frac{{x_{i} }}{RT}\left( {\frac{\partial \gamma }{{\partial x_{i} }}} \right)_{{T,p,n_{J} \ne i}} = \frac{{a_{i} }}{RT}\left( {\frac{\partial \gamma }{{\partial a_{i} }}} \right)_{{T,p,n_{j \ne i} }} $$

According to the definition of the mole fraction of component *i* in the mixtures: $x_{i} = C_{i} /\sum {C_{i} }$, and when $C_{i} < < \sum {C_{i} }$, Eq. 9 can be rewritten in the form:

10 $$ \frac{{C_{i} }}{RT}\left( {\frac{\partial \gamma }{{\partial C_{i} }}} \right)_{{T,p,n_{j \ne i} }} = \frac{{a_{i} }}{RT}\left( {\frac{\partial \gamma }{{\partial a_{i} }}} \right)_{{T,p,n_{j \ne i} }} $$

From the above considerations it results that each form of the Gibbs isotherm equation can be used for calculation of the surface excess concentration of a given surface active agent but under proper conditions.

### Prediction of Solution Surface Tension

The solution surface tension can be predicted on the basis of the activities of the solution components in the bulk phase and surface region, as well as their molar surface area. The relationships between surface tension of nonelectrolite solutions and the activity of their components can be obtained by taking into account the definition of chemical potentials of the component *i* in the bulk and surface phases. From Eqs. 3a and 5a for aqueous solutions of short chain alcohols the Sprow and Prausnitz equations [31] assume the following forms:

11 $$ \gamma_{R} = \gamma_{W} + \frac{RT}{{\omega_{W} }}\ln \frac{{a_{W}^{S} }}{{a_{W}^{B} }} $$

and

12 $$ \gamma_{R} = \gamma_{A} + \frac{RT}{{\omega_{A} }}\ln \frac{{a_{A}^{S} }}{{a_{A}^{B} }} $$

where *γ* refers to the surface tension of solution (*R*), water (*W*) and alcohol (*A*), while *ω* refers to molar surface area of water (*W*) and alcohol (*A*).

Treating alcohol as a solute but not as co-solvent, the surface tension of an aqueous solution of short chain alcohols can be predicted also on the basis of the equation resulting from Eqs. 4a and 6a. This equation has the form:

13 $$ \gamma_{R} = \gamma_{A} + \frac{RT}{{\omega_{A} }}\ln \frac{{a_{A}^{*S} }}{{a_{A}^{*B} }} $$

Of course, for dilute solutions in Eq. 13 the mole fraction of alcohol in the surface and bulk phase can be used instead of the activity.

### Standard Gibbs Energy of Adsorption

The tendency of surface active agents to adsorb at a water–air or water–oil interface can be determined on the basis of the standard Gibbs energy of adsorption ($\Updelta G_{\text{ads}}^{ 0}$). In the literature there are many different calculation methods for $\Updelta G_{\text{ads}}^{ 0}$. For this purpose the commonly used equation is that of Langmuir [1] modified by de Boer [32], which has the form [1, 29]:

14 $$ \frac{{A_{0} }}{{A - A_{0} }}\exp \frac{{A_{0} }}{{A - A_{0} }} = \frac{C}{w}\exp \left( {\frac{{ - \Updelta G_{\text{ads}}^{0} }}{RT}} \right) $$

where *w* is the number of water molecules in 1 dm^3^, *A* is the area occupied per molecule at the interface and *A*_0_ is the “excluded area”, i.e., the area of the interface unavailable to one molecule due to the presence of another.

In the case of aqueous solutions of alcohols, Eq. 14 can be used if they are treated as co-surfactants and their activity coefficient is assumed to be equal to unity for dilute solutions.

Because it is sometimes difficult to obtain real results of the surface excess concentration of the surface active agent at the water–air interface for low concentrations in the bulk phase, Rosen and Aronson [33] proposed another equation for calculation of $\Updelta G_{\text{ads}}^{ 0}$ on the basis of data concerning the surface active agent concentration in the bulk phase corresponding to their saturated monolayer at the water–air interface. This equation for nonionic surface active agents has the form:

15 $$ \Updelta G_{\text{ads}}^{ 0} = RT\ln a_{i}^{*} - \Uppi \omega_{i} $$

where $\omega_{i} = A_{m} \cdot N$ (*N* is the Avogadro number, and *A*_*m*_ is the minimal area of the surface active agents per molecule calculated from $\Upgamma_{m}^{{}}$). It should be noted that Eq. 15 was proposed for a solute for which the standard chemical potential is defined for $f_{i}^{*} \to 1$ and *x*_i_ → 0. Thus, in the case of the alcohol, if the activity is determined on the basis of its partial pressure from the Laar equation [28] as can be found in the literature [25, 34], as a first approximation the mole fraction of alcohol in the bulk phase may be used instead of the activity for the standard Gibbs energy of adsorption calculation.

Sometimes the equation based on a negative logarithm from surfactant concentration is useful, for which the surface tension of the solvent is reduced by 20 mN·m^−1^ (*pC*_20_). In this case Eq. 15 assumes the form [1, 13, 35]:

16 $$ \Updelta G_{ads}^{0} = - 2.303RT(pC_{20} + K) $$

where *K* is a constant which, for a given *T*, depends on the molar surface area of the surface active agents. Equation 16 should be fulfilled for nonionic surface active agents. For such agents Gamboa and Olea [35] proposed that *K* = 1.3. Of course, Eq. 16 should be fulfilled for alcohols, if we assume that the concentration at which they reduce the water surface tension by 20 mN·m^−1^ alcohols activity in the bulk phase ($a_{i}^{*}$) does not greatly differ from their mole fraction, i.e., that the solution behaves ideally.

## Experimental

### Materials

Methanol (99 % purity), ethanol (99 % purity) and propanol (99 % purity) were obtained from SIGMA-ALDRICH and purified by fractional distillation in the presence of magnesium with iodine as an activator [8] and kept over molecular sieves. Aqueous solutions of alcohols were prepared using doubly distilled and deionized water (Milli-Q system) which had an internal specific resistance of 18.2 MΩ. The purity of water was additionally monitored by surface tension measurements before preparing the solutions. The alcohol molar fraction varied from 0 to 1.

### Measurements

The equilibrium surface tension ($\gamma_{LV}$) was measured at 293 K using a Krüss K9 tensiometer, by the platinum ring detachment method (du Nouy’s method). Before surface tension measurements, the tensiometer was calibrated by using water ($\gamma_{LV}$ = 72.8 mN·m^−1^) and methanol ($\gamma_{LV}$ = 22.5 mN·m^−1^), respectively. The measured surface tension values were corrected according to the procedure of Harkins and Jordan [36]. The ring was cleaned with distilled water and heated to red color with a Bunsen burner before each measurement. In all cases more than ten successive measurements were carried out. The standard deviation depending on the region of alcohol concentration was in the range from ±0.1 to ±0.25 mN·m^−1^. The measurement temperature was controlled by a jacketed vessel joined to a thermostatic water bath. All experiments were done at 293 K within ±0.1 K.

The density was measured with an U-tube densitometer (DMA 5000 Anton Paar) at a constant temperature of 293 K. The accuracies of the thermometer and the density measurements are ±0.01 K and ±0.005 kg·m^−3^, respectively. The precisions of the density and temperature measurements given by the manufacturer are ±0.001 kg·m^−3^ and ±0.001 K. The densitometer and viscosimeter were calibrated regularly with distilled and deionized water and methanol.

All viscosity measurements of the aqueous solution of studied surfactants were performed with the Anton Paar viscosimeter (AMVn) at 293 ± 0.1 K with the precision of 0.0001 mPa·s for dynamic viscosity and 0.0001 mm^2^·s^−1^ for kinematic viscosity, respectively, with an uncertainty of 0.3 %.

The size of the alcohol aggregates was determined by using a Zetasizer Nano (Malvern, UK).

## Results and Discussion

### Isotherm of Surface Tension and Activity

**Fig. 1:**
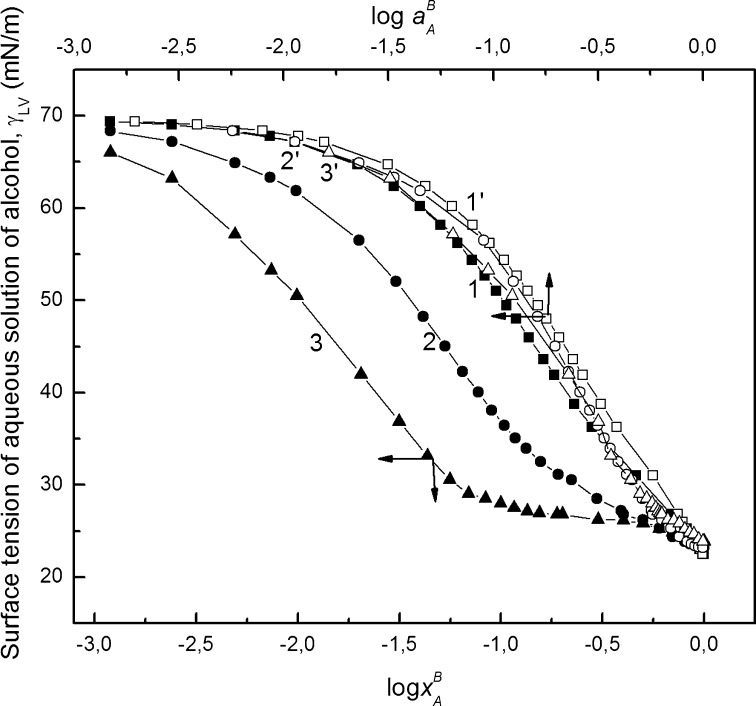
A plot of the surface tension of alcohol (*γ*_*LV*_) versus logarithm (base 10) of alcohol molar faction in the bulk phase ($x_{A}^{B}$) (curves 1–3) and the logarithm of alcohol activity ($a_{A}^{B}$) (curves 1′–3′). Curves 1, 1′ correspond to methanol, curves 2, 2′ to ethanol and curves 3, 3′ to propanol. The plot of surface tension data is taken from [42]

Multiple studies by several independent experimental methods indicate that alcohol molecules aggregate in aqueous solutions [2, 10, 13, 15, 37–39]. This is also reflected in surface tension isotherms. On these isotherms break points were found which suggest that at a given alcohol concentration, aggregation of its molecules takes place [2, 12–14, 24–26]. The data presented in Fig. 1 (Supplementary Table S1) confirms the presence of break points on the $\gamma_{LV} - \log x_{A}^{B}$ curves ($\gamma_{LV}$ is the surface tension of aqueous solution of alcohols and $x_{A}^{B}$ is their mole fraction). However, the break point for propanol is most evident (Fig. 1, curve 3), but it is somewhat doubtful for methanol (Fig. 1, curve 1).

The values of the mole fraction corresponding to the aggregation of methanol, ethanol and propanol determined on the basis of the surface tension isotherms are equal to 0.279, 0.167 and 0.07, respectively (Table 1). These values for ethanol and propanol are close to those determined by Kahlweit et al. [11] at 25 °C, but are lower than those obtained by Zana and Eljebari [10], and Hayashi and Udagawa [12]. In the case of methanol the molar fraction corresponding to the break point on the curve $\gamma_{LV} - \log x_{A}^{B}$ (Fig. 1, curve 1) is considerably lower than that of the operational critical mole fraction of methanol aggregation determined by Zana and Eljebari [10]. Because of this disagreement we carried out measurements of the aggregate size of methanol, ethanol and propanol by using a Zetasizer Nano (Malvern, UK) in the range of molar fractions in which the presence of alcohols in aggregated form can be expected on the basis of our surface tension, density and viscosity results and the literature data. Additionally we carried out such measurements for “pure” alcohols, their dilute aqueous solutions and solutions at alcohol molar fraction equal to 0.5. On the basis of these measurements and the partial alcohol molar volume corresponding to a given alcohol concentration, the aggregation number of its molecules was calculated. For these calculations, spherical aggregates were assumed. Evidence of alcohol aggregates was obtained, at first approximation, in the range of molar fraction corresponding to the break points on the surface tension, density and viscosity isotherms, and above the alcohol molar fraction $X_{A}^{B} = 0.5$; the size of the aggregates was not identified by the Zetasizer Nano measurements (Supplementary Table S2). From light scattering measurements by Zetasizer Nano it appears that probably, at most trimers of methanol associated by hydrogen bonds could be present in the solutions. In the case of ethanol it is possible that aggregation of its molecules takes place not only by interaction of hydrogen bonds but also of hydrophobic ones because ethanol aggregates can include more than 5 molecules. It appears that the aggregation number of ethanol increased slightly from 5 to 9 molecules in the range of its molar fraction, including all literature data, at which ethanol aggregation was expected. The aggregation number of ethanol agrees with that found in the literature [24]. The biggest aggregates are formed by propanol molecules which the size increase in the range of its molar fraction from 0.07 to 0.1. At mole fraction equal to 0.07 eleven-member aggregates can be formed (Supplementary Table S2), which is in accordance with the Roney et al. [15] study. They stated that the hydrophobic association of propanol is independent of the hydrogen bonding state and results in the formation of an approximately a 10-member micelle structure centered around the propanol chains. However, at concentration of 0.1, close to that proposed by Zana and Eljebari [10], the biggest aggregates are formed, including somewhat more than 40 molecules. The possibility of forming aggregates of alcohol and water separately and alcohol–water aggregates in the bulk phase and at the water–air interface should be demonstrated by the alcohol activity in the bulk and surface phase. However, it is obvious that break points on $\gamma_{LV} - \log_{10} a_{A}^{B}$ curves were not detected. Moreover, the changes of the surface tension of aqueous solutions of methanol, ethanol and propanol as a function of logarithm of their activity in the bulk phase ($a_{A}^{B}$), determined on the basis of the Laar equation [28], differ only slightly among the alcohols (Fig. 1). From the data presented in Fig. 1 it can be expected that the activity of each alcohol in the surface monolayer, determined from Eq. 12 on the assumption that the molar area does not depend on the type of alcohol (1.26 × 10^5^ m^2^·mol^−1^) [34] and its concentration, should be the same as that calculated from the following equation [40]:

17 $$ \pi = \pi_{\hbox{max} } a_{A}^{S} $$

where *π* is the difference between water and aqueous solution surface tensions, and *π*_max_ is the difference between the water and alcohol surface tensions.

**Table 1:** The values of the critical alcohol aggregation concentration

Determined from	Critical concentration of aggregation (in mole fraction)		
	Methanol	Ethanol	Propanol
Surface tension	0.279 ± 0.015	0.167 ± 0.011	0.07 ± 0.01
Density	0.224 ± 0.012	0.149 ± 0.013	0.074 ± 0.009
Viscosity	0.318^a^ ± 0.045	0.134^a^ ± 0.014	0.07^a^ ± 0.008
	0.337^b^ ± 0.039	0.148^b^ ± 0.011	0.068b ± 0.009
Average	0.29	0.15	0.07

^a^Determined from dynamic viscosity

^b^Determined from kinematic viscosity

However, for all alcohols, there are differences between the activities calculated from Eqs. 12 and 17 (Supplementary Fig. S1). This means that the molar surface area of methanol, ethanol and propanol depends on their concentration and/or the activity of alcohols in the bulk phase, calculated from the Laar equation [28] on the basis of partial pressure of alcohols over aqueous solutions, and does not show the real interaction of alcohol molecules in aqueous solutions. To explain this problem we calculated the activity of alcohol in the surface layer on the basis of Eq. 11. Because in our case the activity of water ($a_{W}^{B}$) and alcohol were defined in the symmetrical way (the $f_{W}^{B}$ and $f_{W}^{A}$ are approaching unity as $x_{W}^{B} \to 1$ and $x_{W}^{A} \to 1$, respectively), the activity of water used in Eq. 11 is equal to 1 − $a_{A}^{B}$, and the activity of alcohol in the surface layer is equal 1 − $a_{W}^{S}$. For all calculations using Eq. 11 it was assumed that the molar area of water is constant for all solutions studied, equal to 0.6023 × 10^5^ m^2^·mol^−1^. From Supplementary Fig. S1 it appears that the activities of the alcohols calculated in this way are closer to those determined from Eq. 17 than those calculated from Eq. 12. This suggests that the structure of water at the solution–air interface is practically independent of the type of alcohol and their concentration, which is in accordance with low-frequency Raman studies of various concentrations of aqueous solution of propanol carried out by Roney et al. [15]. From these studies it seems that the water structure is largely unaffected except for a small amount of disruption at the interface between the bulk solvent and propanol clusters, with the formation of small water clusters at the interface with bulk-like solvent that interact with hydroxyl groups at the end of the propanol chain [15]. It is possible that the same phenomena occurs in ethanol solution, but it is unlikely in those of methanol. The agreement between the activity values calculated from Eq. 11 [31] and 17 does not explain exactly where the molar area of alcohols is equal to 1.26 × 10^5^ m^2^·mol^−1^ and does not depend on their concentration in solution. Assuming that these conditions are really fulfilled, it is possible to calculate the activity of alcohols in the bulk phase from Eq. 12 taking into account the values of alcohol activity obtained from Eq. 17. As is seen from Supplementary Fig. S2, there are differences between the activity of an alcohol calculated from the Laar [28] equation and Eq. 11 [31]. These differences are the smallest for methanol over the whole range of its concentration in solution. If the values of alcohol activities calculated from Eq. 11 [31], on the assumptions mentioned above, are the real activities of alcohols determined on the basis of Eq. 12, then they should be the same as those calculated from Eq. 17. The alcohol activity determined on the basis of Eq. 12, with the assumption that the sum of the activities of water and alcohol in the bulk phase and surface region are equal to unity for all alcohols, is nearly the same as that calculated from Eq. 17. However, this agreement suggests that it is possible that the alcohol activities calculated from the Laar equation are somewhat different from the real ones, but it is not impossible that the molar area of alcohol changes as a function of its concentration in solution. This change is probably the smallest for methanol because the difference between the activities calculated from the Laar equation [28] and Eq. 11 [31] are the smallest. It is interesting that the dependence of the surface tension on the logarithm of the activity calculated on the basis of Eq. 11 [31] can, practically, be described by one $\gamma_{LV} - \log_{10} a_{A}^{B}$ curve for all alcohols studied (Supplementary Fig. S3). However, no break points are observed on this curve.

### Excess of Alcohol Surface Concentration

According to the general form of Eq. 2 and the definition of the chemical potential, it is possible to determine the correct values of the surface excess of alcohol concentration at the solution–air interface if we take into account its activity in the bulk phase, which is sometimes negligible. As was mentioned above, using the activity of alcohol defined in both ways, it is possible to obtain the same values of the surface excess of alcohol concentration Γ. However, in our case it is impossible to establish the alcohol activity on the assumption that $f_{i}^{*} \to 1$ when $x_{i} \to 0$. On this assumption, in a narrow range of alcohol concentration the values of Γ determined by using the mole fraction of alcohol and its activity defined in the second way should be the same.

**Fig. 2:**
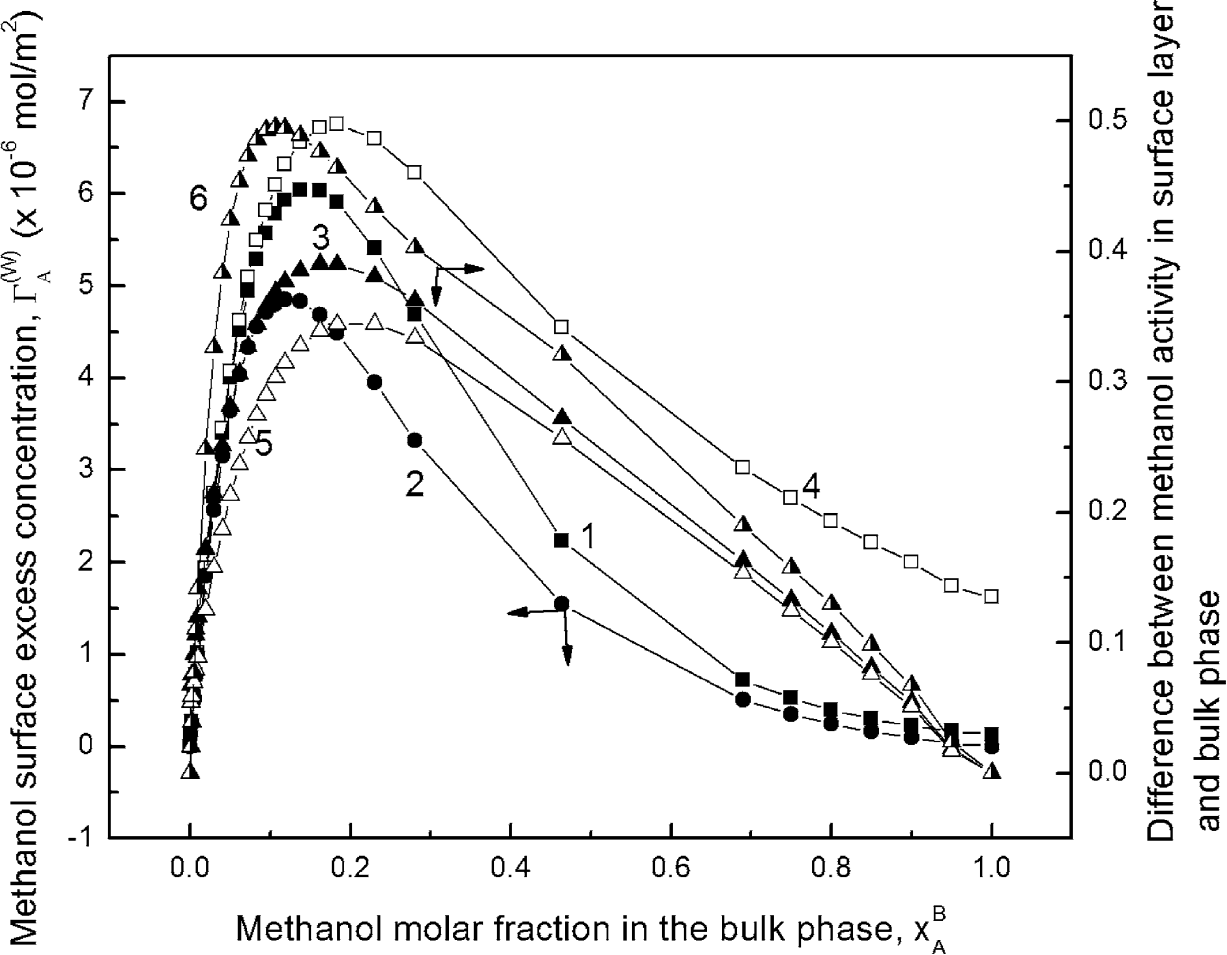
A plot of methanol surface excess concentration ($\Upgamma$) (curves 1–3) and differences between the methanol activity in the surface layer at solution–air interface ($a_{A}^{S}$) and in the bulk phase ($a_{A}^{B}$) (curves 4–6) versus methanol molar fraction in the bulk phase ($x_{A}^{B}$). Curves 1 and 2 correspond to the methanol surface excess concentration calculated from the Gibbs equation by using $x_{A}^{B}$ and $a_{A}^{B}$, respectively, curve 3 to the methanol surface excess concentration calculated from Eq. 18 and curves 4–6 correspond to $a_{A}^{S}$–$a_{A}^{B}$ ($a_{A}^{S}$—determined on the basis of Eqs. 11, 17 and 12), respectively

This is confirmed by the data presented in Fig. 2 and Supplementary Figs. S4a and S4b. In the range of alcohol concentrations at which the values of Γ calculated on the basis of $x_{A}^{B}$ and $a_{A}^{B}$ fulfill Eq. 9, the aqueous solution of alcohol according to the definition can be treated as ideal. This concentration range decreases from methanol to propanol and is even narrower for methanol, as reported by Omelyan et al. [41] ($x_{A}^{B}$ = 0.03). In contrast to Yano [25], the maximum of alcohol adsorption does not correspond to an ideal solution and depends on the type of alcohol and is smaller than 8 × 10^−6^ mol·m^−2^ (Supplementary Table S3). The value of the alcohol molar fraction at which this maximum occurs is also smaller than determined by Yano [25] and Raina et al. [24]. The biggest values of $x_{A}^{B}$ for a given alcohol were mentioned by Raina et al. [24]. In our opinion the differences between the values of $x_{A}^{B}$ observed by us and other investigators may result more from the method of calculating $\partial \gamma_{LV} /\partial a_{A}^{B}$ or $\partial \gamma_{LV} /\partial \log a_{A}^{B}$ or of the alcohol activity determination in the bulk phase than from the results of surface tension measurements. If the activity of alcohol determined from Eq. 12 for ω = 1.26 × 10^5^ m^2^·mol^−1^ is used for calculation of the Gibbs surface excess of alcohol then the maximum of Γ for all alcohols is the same and close to 6 × 10^−6^ mol·m^−2^. Generally, the Γ values determined on the basis of mole fraction of alcohol are lower than those of the activity and the maximum on the isotherm of adsorption is moved towards a higher concentration of alcohol. This means that for calculation of alcohol surface excess concentration the activity should be taken into consideration. However, the Gibbs surface excess concentration is inconvenient, since it is readily physically understood only for small concentrations of strongly adsorbable substances and the calculated excess concentration at a mole fraction of the substrate *x*_i_ → 1 is higher than zero. For this reason Guggenheim and Adam [42] defined a number of other surface excess amounts. Among other things, according to them the Gibbs surface was chosen so that the number of moles of a given substance in the real system could be compared with that of the same substance in the reference system with the same total volume.

The relationship between the Gibbs surface excess $\left( {\Upgamma_{A}^{(W)} } \right)$ and that of Guggenheim and Adam $\left( {\Upgamma_{2}^{(V)} } \right)$ [42] for aqueous solutions of alcohol is as follows:

18 $$ x_{W}^{B} \Upgamma_{A}^{(W)} = \frac{{\overline{V} }}{{V_{W} }}\Upgamma_{A}^{\left( V \right)} $$

where $\overline{V} = x_{W}^{B} V_{W} + x_{A}^{B} V_{A}$ is the average molar volume of the solution.

Introducing the values of $\Upgamma_{A}^{(W)}$ determined on the basis of the Gibbs equation into Eq. 18, the Guggenheim–Adam surface excess concentration was calculated.

**Fig. 3:**
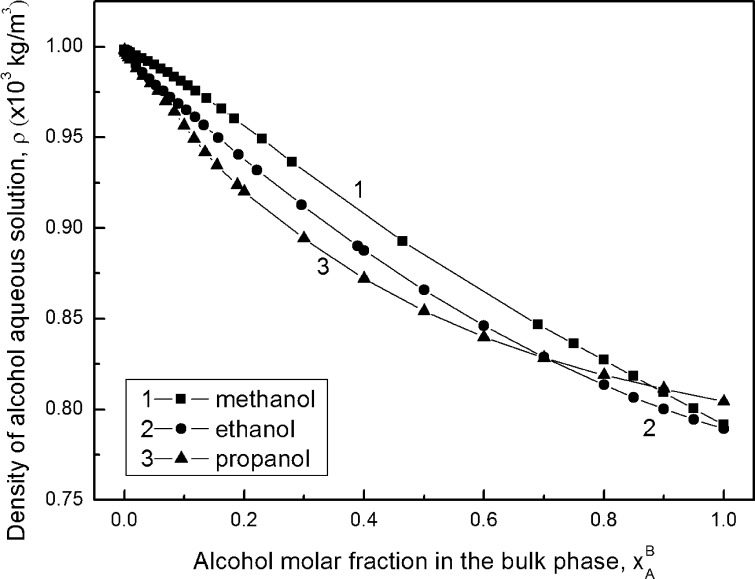
Dependence of the density of alcohol aqueous solutions (*ρ*) on its molar fraction in the bulk phase ($x_{A}^{B}$). Curves 1, 2, and 3 correspond to methanol, ethanol and propanol, respectively

**Fig. 4:**
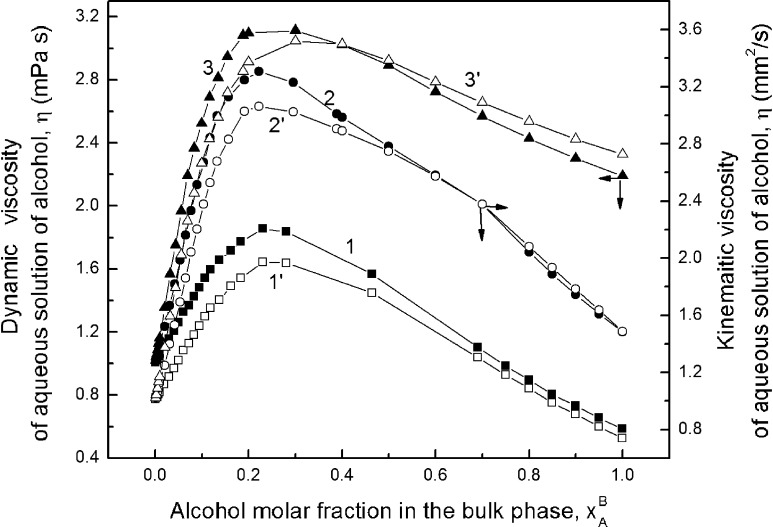
Dependence of the viscosity of aqueous alcohol solutions (*η*) on its molar fraction in the bulk phase ($x_{A}^{B}$). Curves 1, 2 and 3 correspond to the dynamic viscosities of methanol, ethanol and propanol, respectively, and curves 1′, 2′ and 3′ to their kinematic viscosity

Of course, the values of alcohol surface excess concentration calculated from Eq. 18 are close to those calculated from the Gibbs equation only in the range of alcohol concentration narrower than that corresponding to the maximum on the adsorption isotherm as would be expected. According to the studies of Yano [25], on all Gibbs and Guggenheim–Adam adsorption isotherms only one maximum of Γ was observed. This maximum occurred at the same concentration of propanol in solution for the Gibbs and Guggenheim–Adam isotherms (Supplementary Fig. S4b). For ethanol the maximum of $\Upgamma_{A}^{\left( V \right)}$ is at the same concentration as the $\Upgamma_{A}^{(W)}$ if determined on the basis of $x_{A}^{B}$ (Supplementary Fig. S4a). In the case of methanol (Fig. 2) the maximum of $\Upgamma_{A}^{\left( V \right)}$ occurs at a lower concentration than $\Upgamma_{A}^{(W)}$. But if we take into account the alcohol activity in the bulk phase of the solution calculated from Eq. 11 in the way mentioned above, then two maxima on both Gibbs and Guggenheim–Adam isotherms are observed, as suggested by Lavi and Marmur [26] (Supplementary Figs. S5, S6). From Supplementary Fig. S5 it is clearly seen that these maxima appear at different molar fractions for each alcohol studied, and the different molar fractions depend on the kind of isotherm. It is very interesting that if we express the Gibbs and Guggenheim–Adam adsorption isotherms in the form of dependence between the alcohol surface excess concentration and its activity in the bulk phase then the second maximum on the isotherm of Guggenheim–Adam depends on the kind of the alcohol. However, the isotherm of Gibbs for all alcohols has the same shape, the second maximum corresponds to the activity equal to 0.5, and the differences between the $\Upgamma_{A}^{(W)}$ values for methanol, ethanol and propanol are small. It should be expected that at alcohol activity equal to 0.5 aggregates of alcohol are formed because this activity at first approximation corresponds to the molar fraction at which some inflection points are observed (Figs. 1, 3, 4) on the isotherms of the surface tension, density and viscosity of aqueous solutions of alcohols. It is possible that two maxima on the Gibbs adsorption isotherm shown in Supplementary Fig. S6 can point to different tendencies to adsorb single molecules of alcohols, dimers and larger aggregates of alcohols at the solution–air interface. The surface excess of alcohol concentration should be reflected by a difference between the alcohol activity in the surface layer and bulk phase. This is confirmed by the data presented in Fig. 2 and Supplementary Figs. S4a and S4b. The shape of the curves representing the changes of the difference between alcohol activity in the surface layer and bulk phase as a function of alcohol mole fraction is somewhat similar to the isotherm of Guggenheim–Adam. Of course, the maxima on these curves are slightly shifted in comparison to those on the Guggenheim–Adam adsorption isotherm. However, these changes confirm the relationship between the form in which alcohol is present in the bulk phase and its tendency to adsorb at solution–air interface.

### The Standard Gibbs Energy of Alcohol Adsorption

The standard Gibbs energy of adsorption ($\Updelta G_{\text{ads}}^{ 0}$) is a measure of alcohol’s tendency to adsorb at the interface. Because the dilute aqueous alcohol solutions can be treated as ideal, as confirmed above, it is possible to calculate the standard Gibbs energy of alcohol adsorption from Eq. 14 on the basis of the alcohol surface excess concentration calculated from the Gibbs isotherm equation corresponding to a low alcohol concentration in the bulk phase. The $\Updelta G_{\text{ads}}^{ 0}$ values calculated from Eq. 14 indicate that the differences between the standard Gibbs energy of adsorption of methanol, ethanol and propanol are nearly the same and correspond to the work of transfer of one mole of –CH_2_– group from the bulk phase to the surface region determined from the data obtained for classical surfactants [1].

It is interesting that the standard Gibbs energy of methanol adsorption is close to that of two moles of –CH_2_ groups, which is in accordance with our above considerations. To calculate the $\Updelta G_{\text{ads}}^{ 0}$ from Eq. 14, correct data of the surface tension at low concentrations of surface active agents are needed. As known, it is sometimes difficult to obtain good data at this concentration of surface active agents; therefore, Eq. 15 is used for $\Updelta G_{\text{ads}}^{ 0}$ calculation, based on the data corresponding to saturated surface monolayer at the solution–air interface. The application of this equation to classical surfactants is understandable because it is possible to assume that the surfactant activity corresponding to the saturated monolayer is practically equal to its molar fraction. However, for such surface active agents as alcohols the application of Eq. 15 is more complicated. It was stated earlier [43] that using Eq. 15 for the $\Updelta G_{\text{ads}}^{ 0}$calculation for methanol, ethanol and propanol, the same values are obtained for all alcohols (Table 2), if in this equation the alcohol activity determined from the Laar equation [28] (on the basis of partial pressure of the alcohol solution) is used [27, 44]. On the other hand, it should be remembered that the activity of alcohols can be defined in two ways as mentioned above. Thus, if Eq. 9 is fulfilled the $\Updelta G_{\text{ads}}^{ 0}$ for short chain alcohols can be calculated on the basis of the mole fraction of alcohol corresponding to saturated monolayer of alcohol at the solution–air interface. The values of $\Updelta G_{\text{ads}}^{ 0}$ calculated in this way from Eq. 15 are, for each alcohol, somewhat lower than those calculated from the Langmuir equation (Eq. 14) [1]. However, the differences between $\Updelta G_{\text{ads}}^{ 0}$ values for each alcohol studied are similar. This confirms the conclusion drawn above that, at the alcohol concentration corresponding to a saturated monolayer at the solution–air interface, the aqueous solution of alcohol is not ideal, and in such case Eq. 15 gives different values of $\Updelta G_{\text{ads}}^{ 0}$ than those determined form the Langmuir equation (Eq. 14) [1]. Sometimes it is convenient to determine $\Updelta G_{\text{ads}}^{ 0}$ from Eq. 16 [35]. The $\Updelta G_{\text{ads}}^{ 0}$ values calculated from this equation differ considerably from those determined from Langmuir equation (Eq. 14) [1]. This probably resulted from the fact that the constant in Gamboa and Olea’s equation [35] (Eq. 16), equal to 1.3, is not acceptable. Thus, this equation was modified by us and new constants were proposed in this equation. The $\Updelta G_{\text{ads}}^{ 0}$ values calculated from this equation are closer to those obtained from the Langmuir [1] (Eq. 14) and Rosen and Aronson equations [33] (Eq. 15). On the basis of many data for the standard Gibbs energy of adsorption of classical surfactants it was found that a part of this energy corresponding to –CH_2_– groups lies in the range of −3.5 to −3 kJ·mol^−1^. Assuming that the average value is equal to −3.25 kJ·mol^−1^ and that the –CH_3_ group corresponds to two –CH_2_ groups [45], we obtain the $\Updelta G_{\text{ads}}^{ 0}$ values for methanol, ethanol and propanol equal to −6.5, −9.75 and −13 kJ·mol^−1^, respectively. These values are very close to average ones calculated from the Langmuir (Eq. 14) [1], Rosen and Aronson (Eq. 15) [33] and modified Gamboa and Olea equations (Eq. 16) [35].

**Table 2:** Values of the standard Gibbs energy of alcohol adsorption calculated from different equations

Equation	Standard Gibbs energy of alcohol adsorption ($\Updelta G_{\text{ads}}^{0}$ in kJ·mol^−1^)		
	Methanol	Ethanol	Propanol
Eq. 14	−6.26 ± 0.38	−9.28 ± 0.42	−12.41 ± 0.43
Eq. 15	−6.23^a^ ± 0.4	−6.91^a^ ± 0.41	−6.68^a^ ± 0.39
	−7.86^b^ ± 0.45	−10,41^b^ ± 0.49	−13,51^b^ ± 0.5
Eq. 16	−3.75^c^ ± 0.29	−6.14^c^ ± 0.33	−9.39^c^ ± 0.39
	−6.57^d^ ± 0.31	−11.02^d^ ± 0.35	−14.37^d^ ± 0.45
	−6.5	−9.75	−13

^a^Determined on the basis of alcohol activity if *x*_2_ → 1 then *f* → 1

^b^Determined on the basis of *x*_2_ values

^c^Calculated for *K* = 1.3

^d^Calculated for *K* = 2.16, 2.17 and 2.18 for methanol, ethanol and propanol, respectively

### The Volumetric Properties of Alcohols

The changes of the structure of aqueous solutions of alcohols should be reflected in the changes of their density and viscosity (Fig. 3, 4; Supplementary Tables S4, S5). Similar to the isotherms of the surface tension (Fig. 1; Supplementary Table S1) and the viscosity and density isotherms (Fig. 3, 4), it is possible to find some points which correspond to the concentration of alcohol at which aggregation of its molecules occurs, as mentioned above. The concentrations of alcohols corresponding to these points are listed in Table 1. From this table it is seen that the values of this concentration for propanol determined from the isotherms of the surface tension, viscosity and density are practically the same. However, for methanol there are some differences among these values.

Of course, it is possible to obtain more information about the volumetric properties of alcohol from the apparent (*ϕ*_v_) and partial ($\overline{V}_{M}$) molar volumes.

The apparent volume was determined from the following equation [46, 47]:

19 $$ \phi_{V} = \frac{{M_{S} }}{{\rho_{0} }} + \frac{{1000\left( {\rho_{0} - \rho } \right)}}{C} $$

where *M*_S_ is the molecular weight of alcohol, *C* is the concentration of alcohol in mol·cm^−3^ and $\rho_{0}$ is the density of the pure solvent.

The partial molar volume $\overline{V}_{M}$ was calculated from the following equation [48]:

20 $$ \overline{V}_{M} = \frac{{M_{S} }}{\rho }\left[ {1 - \frac{{\left( {100 - c_{p} } \right)}}{\rho }\frac{d\rho }{{dc_{p} }}} \right] $$

The *ρ* data were fit a polynomial of *C*_*p*_ given by:

21 $$ \rho = a + bC_{p} + dC_{p}^{2} $$

where *a*, *b* and *d* are the constants.

The calculated values of apparent molar volume of methanol, ethanol and propanol indicate that, in contrast to Benson and Kiyohara [19] in the case of methanol, no extremum is observed on curves $\varphi_{V} - x_{A}^{B}$ (Supplementary Fig. S7a). However, for ethanol and propanol there are minima in their excess apparent molar volumes. The minimum for ethanol corresponds to $x_{A}^{B}$ = 0.1 which is higher than the mole fraction at which the maximum Gibbs surface excess occurs, being lower than the concentration at which aggregation of ethanol molecules takes place. A minimum of the apparent molar volume is also observed for propanol, but at $x_{A}^{B}$ = 0.06 which is somewhat lower than the concentration of alcohol aggregation. However, this minimum is higher than the molar fraction of propanol at which maximal surface excess occurs. Of course, the minimum of the molar alcohol volume corresponds to the maximum of negative volume excess ($V_{A}^{\text{E}}$). The maximal $V_{A}^{\text{E}}$ of alcohol in aqueous solutions determined by us (Supplementary Fig. S8) for ethanol and propanol agree with that by Benson and Kiyohara [19] and Dethlefsen et al. [20]; however, they appear at different alcohol concentrations.

To explain the change of the excess volumes of alcohols in aqueous solutions let us consider the change of molecular volumes of alcohols on the basis of density measurements of these solutions. A least-squares analysis of the volume of *n*-alkane molecules in the liquid phase, at different temperatures, leads to the conclusion that this volume can be expressed by the simple relation [49]:

22 $$ V = \left( {l + d} \right)\left( {w + d} \right)^{2} $$

where *V* is the volume of the molecule, *l* and *w* are the length and width of the molecule, and *d* is the constant value for a given temperature corresponding to the intermolecular distance.

It appears that the volumes of *n*-alkanes calculated from Eq. 22 for *d* equal 2 Å [49] are nearly the same as those obtained from the density data. Assuming that this equation can be applied, to a first approximation, to *n*-alcohols and taking into account *w* = 2.6 Å [50, 51], *d* = 2 Å [49] and *V* equal to the volume of one molecule of alcohol calculated from the density data, we can calculate the length of the alcohol molecule in the liquid state. The values of *l* calculated in this way for methanol, ethanol and propanol are lower than those given by Raina et al. [24] (Supplementary Table S6) determined on the basis of the particular bond lengths. However, if we assume the minimal possible value of *d* = 1.58 Å [52], the calculated molecule lengths of methanol, ethanol and propanol are close to those of the alcohols obtained from summarizing the distance between H–C, C–C, C–O and O–H [53]. The adsorption data of alcohol on solid surface and at the water–air interface indicate that the area occupied by one alcohol molecule in a vertical orientation is in the range 20–21 Å^2^ [1, 29]. This is in agreement with the value calculated from Eq. 22 for *w* = 2.6 Å and *d* = 2 Å (21.16 Å^2^), but if we assume the length of alcohol molecule is equal to the value given by Raina et al. [24] then the surface area is equal to 17.47 Å^2^. Thus, the question is which distance between alcohol molecules is more realistic in the bulk phase. It is known that molecules of short chain alcohols can be associated by hydrogen bonding. In such cases the average molecule length which results from its volume in the bulk phase, determined from the density data, is lower than that calculated on the basis of the distance between the particular atoms in the molecule; therefore, the distance between molecules equal to 2 Å is more justified than that based on *d* = 1.58 Å. If so, then the contraction of the molar volume of alcohol in water can result from possible changes of the *d* value from 2 to 1.58 Å. The minimal and maximal values of the mole volume of alcohol calculated from Eq. 22 on the basis of these data are presented in Supplementary Table S6. Comparing these values with apparent and partial mole volumes calculated from the density data, we can state that the minimal molar volume of methanol obtained from the density data for $x_{A}^{B} \to 0$ is nearly the same as calculated from Eq. 22. It is practically impossible that a minimum of excess volume could occur for methanol as suggested by some investigators. In the case of ethanol and propanol the minimal values of the mole volume calculated from Eq. 22 are lower than the minimal ones determined from the density and the existence of a maximal excess volume is therefore justified. From comparison of the molar volume of alcohol calculated from the density data and Eq. 22 it seems that the excess volume of the alcohol in aqueous solution results from the change of the intermolecular distance, which is connected with aggregation of alcohol molecules and the number of alcohol molecules joined by hydrogen bonds and/or by hydrophobic interactions between the alcohol chains.

**Fig. 5:**
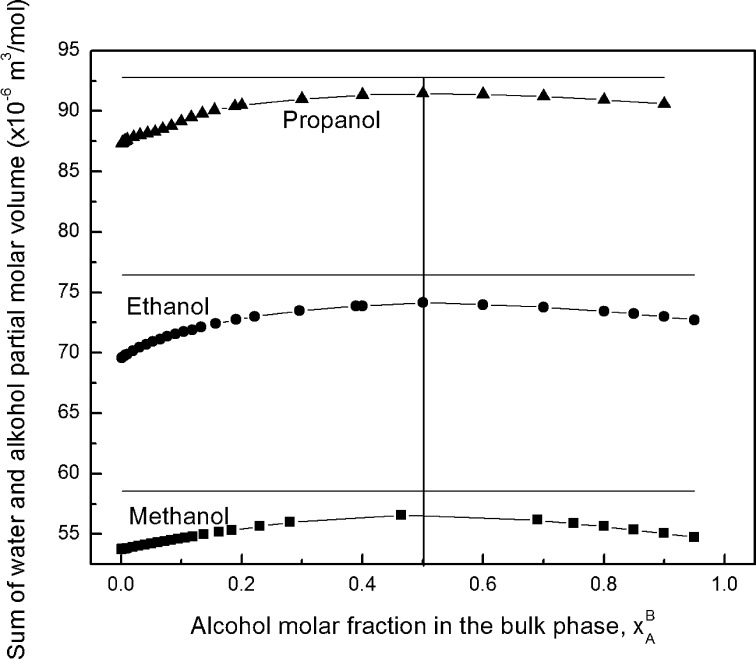
A plot of the sum of water and alcohol partial molar volumes against the alcohol molar fraction in the bulk phase ($x_{A}^{B}$)

Changes of the apparent molar volumes of the alcohols as a function of mole fraction indicate that aqueous solutions of alcohols are nonideal in a wide concentration range as was mentioned above. Nonideality of solutions is, among other things, reflected in changes of the excess volume of solution in comparison to ideal mixtures of the same components. To calculate this excess, first the partial molar volume of water was calculated (Supplementary Fig. S9). Supplementary Fig. S9 shows that if $x_{W}^{B} \to 0$ then the partial molar volume of water in solutions of methanol and ethanol approaches 14 × 10^−6^ m^3^·mol^−1^ [28], which is in accordance with commonly assumed values. In the case of aqueous solutions of propanol the partial molar volume of infinitely dilute water in propanol is equal to 15.6 × 10^−6^ m^3^·mol^−1^. Taking into account the alcohol and water partial molar volumes at a given alcohol concentration, the sum of these volumes was calculated and is presented in Fig. 5.

From this figure it is seen that the shape of the curves showing the changes of the sum of the partial molar volumes of alcohol and water as a function of alcohol mole fraction in the bulk phase is similar for each alcohol solutions and the maximum corresponding to $x_{A}^{B}$ = 0.5 is observed on these curves. This maximum indicates that there is a minimum in the deviation between the volume of real and ideal solution if the ratio of alcohol molecules to water is 1:1. It is interesting that the changes of the sum of partial polar volumes of water and alcohol as a function of the solution composition is somewhat similar to changes of excess free enthalpy of water and alcohol mixing [54, 55]. This energy is positive and nearly symmetrical about *x*_2_ = 0.5 and depends on enthalpy and entropy effects. Thus, if mixtures of alcohol with water are made, some hydrogen bonds are broken endothermically and new ones are made exothermically, the enthalpy will be the difference between two much larger thermal effects. Simultaneously a loss of entropy takes places. In aqueous solutions of alcohols, alcohol–alcohol, water–water and alcohol–water molecules joined by hydrogen bonds and aggregates of alcohol formed by hydrophobic interactions are present. The measurements of the size of alcohol molecules by the Zetasizer Nano at *x*_2_ = 0.5 indicate that there are not large aggregates of alcohols. Probably dimers of alcohols are present, but also the formation of the alcohol–water hydrogen bonds cannot be excluded. From the thermodynamic properties of water–ethanol mixtures it results that the entropy effect is decisive for the changes of excess Gibbs energy of mixing. Thus a proper order of the associated alcohol and hydrated water molecules at *x*_2_ = 0.5 has a higher contribution to the excess Gibbs energy than disruption and formation of hydrogen bonds in comparison to “pure” water and alcohol. Therefore, probably a minimal excess of the sum of water and alcohol molar volumes is observed at *x*_2_ = 0.5.

## Conclusions

Measurements of the isotherm of surface tension, density, viscosity and the light scattering show clearly that even methanol forms some aggregates, which for ethanol and propanol are like small micelles at some solution concentrations.

In contrast to other investigators, aqueous solution ideality of short chain alcohols in the range of their concentration from zero to that corresponding to the maximal value of alcohol surface excess concentration was not proved, but a dependence between the maximal value of alcohol surface excess concentration at the solution–air interface and kind of alcohol was observed.

Contrary to expectation, the activity of alcohol in the surface monolayer cannot be determined on the basis of the Sprow and Prausnitz equation, taking into account the activity of alcohol in the bulk phase determined from the Laar equation on the basis of partial pressure of alcohol over the solution and the constant value of the molar area of alcohol in the surface layer. However, it is possible to predict the alcohol activity from these equations, taking into account the activity of water in the bulk phase and the constant value of the water molar area.

The standard Gibbs energy of alcohol adsorption at the water–air interface can be determined successfully both by Langmuir and Rosen and Aronson equations only in the case where asymmetric definitions of the activity are taken into account and, if according to this definition, the solution does not deviate from the ideality in the range of the surface active agent concentrations at which saturated adsorbed monolayer is formed.

There is a contraction minimum of the volume of solutions after mixing alcohol with water if the ratio of their molecules is 1:1, which correlate with the maximum of the surface free enthalpy excess of alcohol and water mixing and is close to the minimal enthalpy and entropy effects, indicating that the decisive contribution of the entropy to volume excess of sum of water and alcohol volumes at molar fraction of alcohol equal to 0.5.

## Electronic supplementary material

Below is the link to the electronic supplementary material.

Supplementary material 1 (DOC 33253 kb)

## References

[1] Rosen JM (2004). Surfactants and Interfacial Phenomena.

[2] Zana R (1995). Aqueous surfactant–alcohol systems: A review. Adv. Colloid Interface Sci..

[3] Zana R, Yiv S, Strazielle C, Lianos P (1981). Effect of alcohol on the properties of micellar systems. I. Critical micellization concentration, micelle molecular weight and ionization degree, and solubility of alcohols in micellar solutions. J. Colloid Interface Sci..

[4] Guveli DE, Kayes JB, Davis SS (1979). Hydrodynamic studies of micellar systems of alkyltrimethyl-ammonium bromides and the effect of added 1-alkanols. J. Colloid Interface Sci..

[5] Attwood D, Mosquera V, Rodriguez J, Garcia M, Suarez MJ (1994). Effect of alcohols on the micellar properties in aqueous solution of alkyltrimethylammonium bromides. Colloid Polym. Sci..

[6] Førland GM, Samseth J, Høiland H, Mortensen K (1994). The effect of medium chain length alcohols on the micellar properties of sodium dodecyl sulfate in sodium chloride solutions. J. Colloid Interface Sci..

[7] Tomšič M, Bešter-Rogač M, Jamnik A, Kunz W, Touraud D (2006). Ternary systems of nonionic surfactant Brij 35, water and various simple alcohols: Structural investigations by small-angle X-ray scattering and dynamic light scattering. J. Colloid Interface Sci..

[8] Vogel, A.I.: Preparatyka Organiczna, wyd. 3, WNT, Warszawa (2006)

[9] Franks F, Franks F (1975). The hydrophobic interaction. Water. A Comprehensive Treatise.

[10] Zana R, Eljebari MJ (1993). Fluorescence probing of self-association of alcohols in aqueous solution. J. Phys. Chem..

[11] Kahlweit M, Busse G, Jen J (1991). Adsorption of amphiphiles at water/air interfaces. J. Phys. Chem..

[12] Hayashi H, Udagawa Y (1992). Mixing state of 1-propanol aqueous solutions studied by small-angle x-ray scattering: A new parameter reflecting the shape of SAXS curve. Bull. Chem. Soc. Jpn..

[13] Zdziennicka A, Jańczuk B (2010). Behaviour of cationic surfactants and short chain alcohols in mixed surface layers at water–air and polymer–water interfaces with regard to polymer wettability. I. Adsorption at water–air interface. J. Colloid Interface Sci..

[14] Yoshida K, Yamaguchi T (2001). Low-frequency Raman spectroscopy of aqueous solutions of aliphatic alcohols. Z. Naturforsch..

[15] Roney AB, Space B, Castner EW, Napoleon RL, Moore RB (2004). A molecular dynamics study of aggregation phenomena in aqueous *n*-propanol. J. Phys. Chem. B.

[16] Alavi S, Takeya S, Ohmura R, Woo TK, Ripmeester JA (2010). Hydrogen-bonding alcohol–water interactions in binary ethanol, 1-propanol, and 2-propanol + methane structure II clathrate hydrates. J. Chem. Phys..

[17] Fidler J, Rodger PM (1999). Solvation structure around aqueous alcohols. J. Phys. Chem. B.

[18] Tamaka H, Gubbins K (1992). Structure and thermodynamic properties of water–methanol mixtures: Role of the water–water interaction. J. Chem. Phys..

[19] Benson GC, Kiyohara O (1980). Thermodynamics of aqueous mixtures of nonelectrolytes. I. Excess volumes of water–*n*-alcohol mixtures at several temperatures. J. Solution Chem..

[20] Dethlefsen Ch, Sørensen PG, Hvidt A (1984). Excess volumes of propanol–water mixtures at 5, 15, and 25 °C. J. Solution Chem..

[21] Mikhail SZ, Kimel WR (1963). Densities and viscosities of 1-propanol–water mixtures. J. Chem. Eng. Data.

[22] Mikhail SZ, Kimel WR (1961). Densities and viscosities of methanol–water mixtures. J. Chem. Eng. Data.

[23] McGlashan ML, Williamson AG (1976). Isothermal liquid–vapor equilibria for system methanol–water. J. Chem. Eng. Data.

[24] Raina G, Kulkarni GU, Rao CNR (2001). Surface enrichment in alcohol–water mixtures. J. Phys. Chem. A.

[25] Yano YF (2005). Correlation between surface and bulk structures of alcohol–water mixtures. J. Colloid Interface Sci..

[26] Lavi P, Marmur A (2000). Adsorption isotherms for concentrated aqueous-organic solutions (CAOS). J. Colloid Interface Sci..

[27] Strey R, Viisanen Y, Aratono M, Kratohvil JP, Yin Q, Friberg SE (1999). On the necessity of using activities in the Gibbs equation. J. Phys. Chem. B.

[28] Atkins, P.W.: Chemia Fizyczna, Wyd. I. PWN, Warszawa (2001)

[29] Adamson AW, Gast AP (1997). Physical Chemistry of Surfaces.

[30] Butler JAV (1932). The thermodynamics of the surfaces of solutions. Proc. R. Soc. A.

[31] Sprow FB, Prausnitz JM (1967). Surface thermodynamics of liquid mixtures. Can. J. Chem. Eng..

[32] De Boer JH (1953). The Dynamic Character of Adsorption.

[33] Rosen JM, Aronson S (1981). Standard free energies of adsorption of surfactants at the aqueous solution/air interface from surface tension data in the vicinity of the critical micelle concentration. Colloids Surf..

[34] Zdziennicka A (2009). The adsorption properties of short chain alcohols and Triton X-100 mixtures at the water–air interface. J. Colloid Interface Sci..

[35] Gamboa C, Olea AF (2006). Association of cationic surfactants to humic acid: Effect on the surface activity. Colloids Surf. A.

[36] Harkins WD, Jordan HF (1930). A method for the determination of surface and interfacial tension from the maximum pull on a ring. J. Am. Chem. Soc..

[37] Romero CM, Paéz MS (2006). Surface tension of aqueous solutions of alcohol and polyols at 298.15 K. Phys. Chem. Liq..

[38] Araton M, Toyomasu T, Villeneuve M, Uchizono Y, Takiue T, Motomura K, Ikeda N (1997). Thermodynamic study on the surface formation of the mixture of water and ethanol. J. Colloid Interface Sci..

[39] Vázquez G, Alvarez E, Navaza JM (1995). Surface tension of alcohol + water from 20 to 50 °C. J. Chem. Eng. Data.

[40] Chataraj DK, Birdi KS, Kalder K, Das KP, Mitra A (2006). Surface activity coefficients of spread monolayers of behenic acid salts at air–water interface. Adv. Colloid Interface Sci..

[41] Omelyan I, Kovalenko A, Hirata F (2003). Compressibility of *tert*-butyl alcohol–water mixtures: The RISM theory. J. Theor. Comput. Chem..

[42] Guggenheim, E. A., Adam, N.K.: Thermodynamics of adsorption at the surface of solutions. Proc. Roy. Soc. Lond. A **139**, 218–236 (1933)

[43] Zdziennicka A (2010). Surface behavior of Triton X-165 and short chain alcohol mixtures. Langmuir.

[44] Nord L, Tucker EE, Christin SD (1984). Liquid vapor equilibrium of dilute aqueous solutions of ethanol and 2-propanol. J. Solution Chem..

[45] Jańczuk B, González-Martín ML, Zdziennicka A, Wójcik W, Bruque JM (2002). The critical micelle concentration of alkylammonium chlorides, sodium alkyl sulphates and sulphonates and their surface free energy. Tenside Surf. Deterg..

[46] Kale KM, Zana R (1977). Effect of the nature of the counterion on the volume change upon micellization of ionic detergents in aqueous solutions. J. Colloid Interface Sci..

[47] Boden N, Corne SA, Jolley KW (1987). Lyotropic mesomorphism of the cesium pentadecafluorooctanoate/water system: High-resolution phase diagram. J. Phys. Chem..

[48] Benjamin L (1966). Partial molal volume changes during micellization and solution of nonionic surfactants and perfluorocarboxylates using a magnetic density balance. J. Phys. Chem..

[49] Jańczuk B, Méndez Sierra JA, González-Martín ML, Bruque ML, Wójcik W (1997). Properties of decylammonium chloride and cesium perfluorooctane at interfaces and standard free energy of their adsorption. J. Colloid Interface Sci..

[50] van Oss CJ, Constanzo PM (1992). Adhesion of anionic surfactants to polymer surfaces and low-energy materials. J. Adhesion Sci. Technol..

[51] Jańczuk B, Méndez Sierra JA, González-Martín ML, Bruque JM, Wójcik W (1996). Decylammonium chloride and cesium perfluorooctane surface free energy and their critical micelle concentration. J. Colloid Interface Sci..

[52] van Oss CJ, Giesse RE, Constanzo PM (1990). DLVO and non-DLVO interactions in hectorite. Clays Clay Miner..

[53] Pauling L (1945). The Nature of the Chemical Bond.

[54] Franks F (1973). Water: A Comprehensive Treatise.

[55] Franks F, Ives DJG (1966). The structural properties of alcohol–water mixtures. Q. Rev. Chem. Soc..

