# Supplementary material for: Volumetric and Surface Properties of Short Chain Alcohols in Aqueous Solution–Air Systems at 293 K
Source: J Solution Chem. 2012 Dec 4;41(12):2226–45. doi: 10.1007/s10953-012-9935-z (PMC3521650; doi:10.1007/s10953-012-9935-z)
Supplement: Supplementary file 1 — Supplementary material 1 (DOC 33253 kb) [file 10953_2012_9935_MOESM1_ESM.doc]

Table S1. Values of the surface tension of aqueous solution of methanol, ethanol and propanol ($\gamma_{LV}$) measured at 293 K.

| Methanol | | Ethanol | | Propanol | |
| --- | --- | --- | --- | --- | --- |
| $X_{2}$ [molar fraction] | $\gamma_{LV}$ [mN·m^−1^] | $X_{2}$ [molar fraction] | $\gamma_{LV}$ [mN·m^−1^] | $X_{2}$ [molar fraction] | $\gamma_{LV}$ [mN·m^−1^] |
| 0 | 72.8 | 0 | 72.8 | 0 | 72.8 |
| 0.0012 | 69.37 | 0.0012 | 68.34 | 0.0012 | 66.02 |
| 0.0024 | 69.04 | 0.0024 | 67.21 | 0.0024 | 63.21 |
| 0.0049 | 68.39 | 0.0049 | 64.89 | 0.0049 | 57.13 |
| 0.0073 | 67.75 | 0.0073 | 63.35 | 0.0074 | 53.25 |
| 0.0097 | 67.12 | 0.0098 | 61.87 | 0.0099 | 50.48 |
| 0.0197 | 64.67 | 0.0200 | 56.53 | 0.0205 | 41.96 |
| 0.0298 | 62.36 | 0.0305 | 52.03 | 0.0316 | 36.85 |
| 0.0401 | 60.18 | 0.0415 | 48.23 | 0.0434 | 33.17 |
| 0.0506 | 58.12 | 0.0529 | 45.02 | 0.0559 | 30.57 |
| 0.0614 | 56.18 | 0.0647 | 42.31 | 0.0693 | 29.06 |
| 0.0724 | 54.34 | 0.0771 | 40.03 | 0.0837 | 28.52 |
| 0.0836 | 52.62 | 0.0901 | 38.1 | 0.0994 | 28.02 |
| 0.095 | 51.01 | 0.1036 | 36.47 | 0.1163 | 27.52 |
| 0.1069 | 49.44 | 0.1178 | 35.1 | 0.1346 | 27.15 |
| 0.1188 | 47.99 | 0.1328 | 33.94 | 0.1545 | 26.97 |
| 0.1374 | 45.96 | 0.1568 | 32.53 | 0.1883 | 26.82 |
| 0.1623 | 43.6 | 0.1908 | 31.17 | 0.2 | 26.82 |
| 0.1837 | 41.86 | 0.2217 | 30.57 | 0.3 | 26.23 |
| 0.2301 | 38.77 | 0.2957 | 28.52 | 0.4 | 26.18 |
| 0.2806 | 36.24 | 0.3888 | 27.2 | 0.5 | 25.85 |
| 0.4644 | 31.01 | 0.4 | 26.8 | 0.6 | 25.24 |
| 0.6901 | 26.81 | 0.5 | 26.2 | 0.7 | 24.87 |
| 0.75 | 25.95 | 0.6 | 25.3 | 0.8 | 24.67 |
| 0.8 | 25.26 | 0.7 | 24.42 | 0.9 | 24.12 |
| 0.85 | 24.54 | 0.8 | 23.87 | 1 | 23.87 |
| 0.9 | 23.86 | 0.85 | 23.61 |  |  |
| 0.95 | 22.98 | 0.9 | 23.38 |  |  |
| 1 | 22.5 | 0.95 | 23.3 |  |  |
|  |  | 1 | 23.2 |  |  |

The measurement uncertainties are: from $\gamma_{LV}$ ± 0.1 to$\gamma_{LV}$ ± 0.2 mN·m^−1^ (depending on the alcohol concentration range); *T* ± 0.1 K,

Table S2. The values of the aggregates diameter (*d*) and the aggregation calculated at first approximation on the basis of this diameter.

| Alcohol | $x_{A}^{B}$ [molar fraction] | *d*  [Å] | *l*_A_ |
| --- | --- | --- | --- |
| Methanol | 0.0024 | <6 |  |
|  | 0.1837 | 6.902 | 2.76 |
|  | 0.2301 | 6.958 | 2.8 |
|  | 0.2806 | 6.975 | 2.8 |
|  | 0.5 | <6 |  |
|  | 1 | <6 |  |
| Ethanol | 0.0024 | <6 |  |
|  | 0.1328 | 9.884 | 5.61 |
|  | 0.1568 | 10.43 | 6.56 |
|  | 0.1908 | 11.55 | 8.8 |
|  | 0.5 | 8.487 | 3.35 |
|  | 1 | <6 |  |
| Propanol | 0.0024 | <6 |  |
|  | 0.0693 | 14.24 | 12.9 |
|  | 0.0837 | 20.34 | 37.45 |
|  | 0.0994 | 21.56 | 44.61 |
|  | 0.5 | 10.47 | 4.85 |
|  | 1 | <6 |  |

Table S3. The maximum values of partial volume excess ($V_{\max}^{E}$) of alcohols and water, maximal value of alcohol excess concentration at water-air interface (Γ_max)_, and alcohol molar fraction ($x_{A}^{B}$) corresponding to them.

| Liquid | $V_{\max}^{E}$ [× 10^−6^ m^3^·mol^−1^] | $x_{A}^{B}$ at $V_{\max}^{E}$ [molar fraction] | Γ_max_ (Eq. (9))  [× 10^−6^ mol·m^−2^] | $x_{A}^{B}$ at Γ_max_  [molar fraction] | $x_{A}^{B}$ at $\Gamma_{\max}^{(V)}$ [molar fraction] |
| --- | --- | --- | --- | --- | --- |
| methanol | −5.843^f^ | 0.0012^f^ (without maximum) | 6.04^a^  6.75^b^ | 0.1374^a^  0.1837^b^ | 0.1188^c^ |
|  |  |  | 7.08^d^ | 0.23^d^ |  |
|  | −3.56^g^ | 0.0789^g^ |  | 0.3^e^ |  |
| ethanol | −5.332^f^ | 0.1036^f^ | 5.492^a^  7.264^b^ | 0.0529^a^  0.0771^b^ | 0.0529^c^ |
|  | −5.25^g^ | 0.0647^g^ | 7.62^d^ | 0.11^d^ |  |
|  |  |  |  | 0.2^e^ |  |
| propanol | −6.072^f^ | 0.0559^f^ | 5.15^a^ | 0.0205^a^ | 0.0205^c^ |
|  |  |  | 6.57^b^ | 0.0205^b^ |  |
|  | −6.53^g^ | 0.0368^g^ | 7.15^d^ | 0.04^d^ |  |
|  | −5.86^h^ | 0.0429^h^ |  | 0.1^e^ |  |
| Water-methanol | −4.08^f^  −3.81^g^ |  |  |  |  |
| water-ethanol | −3.99^f^  −4.19^g^ |  |  |  |  |
| water-propanol | −2.5^f^  −2.84^g^ |  |  |  |  |

^a^ – calculated from Eq. 9 on the basis of alcohol molar fraction

^b^ – calculated from Eq. 9 on the basis of alcohol activity

^c^ – calculated from Eq. 18

^d^ – taken from Ref. [25]

^e^ – taken from Ref. [24]

^f^ – determined by us

^g^ – taken from Ref. [19]

^h^ – taken from Ref. [20]

Table S4 Values of the density ($\rho$) of aqueous solution of methanol, ethanol and propanol measured at 293 K.

| Methanol | | Ethanol | | Propanol | |
| --- | --- | --- | --- | --- | --- |
| $X_{2}$ [molar fraction] | $\rho$ [× 10 kg·m^−3^] | $X_{2}$ [molar fraction] | $\rho$ [× 10 kg·m^−3^] | $X_{2}$ [molar fraction] | $\rho$ [x10 kg·m^−3^] |
| 0 | 0.9982 | 0 | 0.9982 | 0 | 0.9982 |
| 0.0012 | 0.998025 | 0.0012 | 0.99759 | 0.0012 | 0.9975 |
| 0.0024 | 0.997845 | 0.0024 | 0.99701 | 0.0024 | 0.9968 |
| 0.0049 | 0.997485 | 0.0049 | 0.99589 | 0.0049 | 0.9954 |
| 0.0073 | 0.99712 | 0.0073 | 0.99481 | 0.0074 | 0.9942 |
| 0.0097 | 0.99674 | 0.0098 | 0.99378 | 0.0099 | 0.9928 |
| 0.0197 | 0.9952 | 0.02 | 0.98974 | 0.0205 | 0.9881 |
| 0.0298 | 0.99356 | 0.0305 | 0.98595 | 0.0316 | 0.984 |
| 0.0401 | 0.9918 | 0.0415 | 0.98244 | 0.0434 | 0.9799 |
| 0.0506 | 0.9899 | 0.0529 | 0.97896 | 0.0559 | 0.9756 |
| 0.0614 | 0.9878 | 0.0647 | 0.97564 | 0.0693 | 0.97 |
| 0.0724 | 0.9857 | 0.0771 | 0.97214 | 0.0837 | 0.9641 |
| 0.0836 | 0.9834 | 0.0901 | 0.96864 | 0.0994 | 0.9566 |
| 0.095 | 0.9809 | 0.1036 | 0.96507 | 0.1163 | 0.9492 |
| 0.1069 | 0.9784 | 0.1178 | 0.96125 | 0.1346 | 0.9418 |
| 0.1188 | 0.9757 | 0.1328 | 0.956709 | 0.1545 | 0.9345 |
| 0.1374 | 0.9714 | 0.1568 | 0.949875 | 0.1883 | 0.9237 |
| 0.1623 | 0.9656 | 0.1908 | 0.940408 | 0.2 | 0.92001 |
| 0.1837 | 0.9604 | 0.2217 | 0.932022 | 0.3 | 0.89421 |
| 0.2301 | 0.949 | 0.2957 | 0.912785 | 0.4 | 0.87206 |
| 0.2806 | 0.9364 | 0.3888 | 0.890276 | 0.5 | 0.85421 |
| 0.4644 | 0.8926 | 0.4 | 0.887695 | 0.6 | 0.83983 |
| 0.6901 | 0.8468 | 0.5 | 0.865863 | 0.7 | 0.82825 |
| 0.75 | 0.83611 | 0.6 | 0.846207 | 0.8 | 0.81892 |
| 0.8 | 0.82719 | 0.7 | 0.828728 | 0.9 | 0.81141 |
| 0.85 | 0.81827 | 0.8 | 0.813425 | 1 | 0.8043 |
| 0.9 | 0.80934 | 0.85 | 0.80659 |  |  |
| 0.95 | 0.80042 | 0.9 | 0.800299 |  |  |
| 1 | 0.7915 | 0.95 | 0.794553 |  |  |
|  |  | 1 | 0.7894 |  |  |

The accuracy of the thermometer and the density measurements are ±0.01 K and ± 0.005 kg·m^−3^, respectively. The precision of the density and temperature measurements given by the manufacturer was ±0.001 kg·m^−3^ and ±0.001K.

Table S5. Values of the dynamic and kinematic viscosity of aqueous solution of methanol, ethanol and propanol ($\eta$) measured at 293 K.

| Methanol | | | Ethanol | | | Propanol | | |
| --- | --- | --- | --- | --- | --- | --- | --- | --- |
| $X_{2}$ [molar  fraction] | $\eta$ dyn  [mPa·s] | $\eta$ kin  [mm^2^·s^−1^] | $X_{2}$ [molar  fraction] | $\eta$ dyn  [mPa·s] | $\eta$ kin  [mm^2^·s^−1^] | $X_{2}$ [molar  fraction] | $\eta$ dyn  [mPa·s] | $\eta$ kin  [mm^2^·s^−1^] |
| 0 | 1.0020 | 1.0038 | 0 | 1.0020 | 1.0038 | 0 | 1.0020 | 1.0038 |
| 0.0012 | 1.0078 | 1.0101 | 0.0012 | 1.0189 | 1.0213 | 0.0012 | 1.0193 | 1.0218 |
| 0.0024 | 1.0192 | 1.0212 | 0.0024 | 1.0344 | 1.0374 | 0.0024 | 1.0420 | 1.0432 |
| 0.0049 | 1.0317 | 1.0348 | 0.0049 | 1.0594 | 1.0637 | 0.0049 | 1.0852 | 1.0889 |
| 0.0073 | 1.0437 | 1.0479 | 0.0073 | 1.0867 | 1.0924 | 0.0074 | 1.1285 | 1.1346 |
| 0.0097 | 1.0552 | 1.0606 | 0.0098 | 1.1196 | 1.1274 | 0.0099 | 1.1610 | 1.1693 |
| 0.0197 | 1.1057 | 1.1153 | 0.02 | 1.2358 | 1.2489 | 0.0205 | 1.3551 | 1.3741 |
| 0.0298 | 1.1562 | 1.1703 | 0.0305 | 1.3692 | 1.3995 | 0.0316 | 1.5675 | 1.5935 |
| 0.0401 | 1.2078 | 1.2263 | 0.0415 | 1.5053 | 1.5326 | 0.0434 | 1.7513 | 1.7927 |
| 0.0506 | 1.2603 | 1.2834 | 0.0529 | 1.6573 | 1.6941 | 0.0559 | 1.9676 | 2.0211 |
| 0.0614 | 1.3264 | 1.3523 | 0.0647 | 1.8147 | 1.8603 | 0.0693 | 2.1917 | 2.2601 |
| 0.0724 | 1.3693 | 1.4020 | 0.0771 | 1.9702 | 2.0435 | 0.0837 | 2.3658 | 2.4553 |
| 0.0836 | 1.4254 | 1.4630 | 0.0901 | 2.1332 | 2.2021 | 0.0994 | 2.5235 | 2.6624 |
| 0.095 | 1.4824 | 1.5250 | 0.1036 | 2.2781 | 2.3772 | 0.1163 | 2.6891 | 2.8347 |
| 0.1069 | 1.5419 | 1.5897 | 0.1178 | 2.4303 | 2.5281 | 0.1346 | 2.8117 | 2.9849 |
| 0.1188 | 1.5953 | 1.6493 | 0.1328 | 2.5713 | 2.6789 | 0.1545 | 2.9465 | 3.1566 |
| 0.1374 | 1.6566 | 1.7076 | 0.1568 | 2.6923 | 2.8320 | 0.1883 | 3.0815 | 3.3081 |
| 0.1623 | 1.7161 | 1.8033 | 0.1908 | 2.7976 | 3.0284 | 0.2 | 3.0951 | 3.3712 |
| 0.1837 | 1.7735 | 1.8594 | 0.2217 | 2.8537 | 3.0631 | 0.3 | 3.1140 | 3.5186 |
| 0.2301 | 1.8552 | 1.9721 | 0.2957 | 2.7816 | 3.0238 | 0.4 | 3.0215 | 3.4971 |
| 0.2806 | 1.8357 | 1.9665 | 0.3888 | 2.5822 | 2.9048 | 0.5 | 2.8913 | 3.3828 |
| 0.4644 | 1.5670 | 1.7566 | 0.4 | 2.5616 | 2.8901 | 0.6 | 2.7240 | 3.2320 |
| 0.6901 | 1.1036 | 1.3050 | 0.5 | 2.3776 | 2.7488 | 0.7 | 2.5694 | 3.0884 |
| 0.75 | 0.9862 | 1.1823 | 0.6 | 2.1937 | 2.5739 | 0.8 | 2.4282 | 2.9555 |
| 0.8 | 0.8975 | 1.0887 | 0.7 | 2.0098 | 2.3769 | 0.9 | 2.3008 | 2.8315 |
| 0.85 | 0.8055 | 0.9879 | 0.8 | 1.7070 | 2.0806 | 1 | 2.1908 | 2.7257 |
| 0.9 | 0.7320 | 0.9079 | 0.85 | 1.5679 | 1.9324 |  |  |  |
| 0.95 | 0.6552 | 0.8207 | 0.9 | 1.4370 | 1.7842 |  |  |  |
| 1 | 0.5850 | 0.7392 | 0.95 | 1.3144 | 1.6361 |  |  |  |
|  |  |  | 1 | 1.2001 | 1.4879 |  |  |  |

The precision of dynamic and kinematic viscosity measurements was 0.0001 mPa·s and 0.0001 mm^2^·s^−1^, respectively and uncertainty 0.3 %.and *T* ± 0.01 K,

Table S6 Values of the maximal and minimal mole volume of alcohol calculated from the density data and from Eq. 22, and length of alcohol molecule.

| Alcohol | $V_{\max}$ [×10^−6^ m^3^·mol^−1^] Eq. 22 | $V_{\min}$ [×10^−6^ m^3^·mol^−1^] Eq. 22 | $V_{\min}$ [×10^−6^ m^3^·mol^−1^] (Fig. S7a – S7c) | $V_{\max}$ [×10^−6^ m^3^·mol^−1^] (Fig. S7a – S7c) | *h*  [Å] |
| --- | --- | --- | --- | --- | --- |
| Methanol | 40.48 | 33.67 | 34.64 | 40.47 | 3.2^a^  3.85^b^  4^c^ |
| Ethanol | 58.36 | 48,51 | 53.03 | 58.35 | 4.61^a^  5.55^b^  5.5^c^ |
| Propanol | 74.72 | 62.19 | 68.64 | 74.7 | 5.91^a^  7.1^b^ |

^a^ – calculated for d = 2Å

^b^ – calculated for d = 1.58Å

^c^ – taken from Ref. [24]


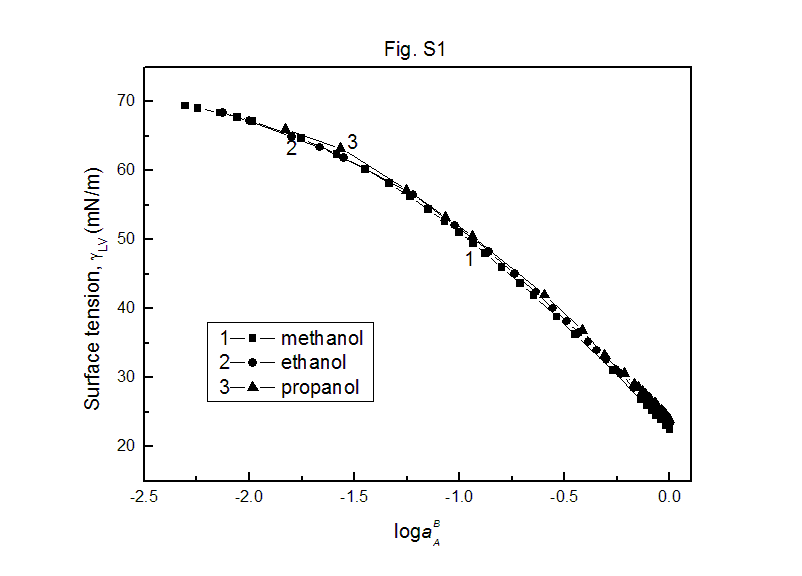


**Fig. S1** A plot of the alcohol surface tension (*γ_LV_*) vs. the logarithm of alcohol activity in the bulk phase ($a_{A}^{B}$) calculated from Eq. 12 for ω = 1.26 × 10^5^ mol·m^−2^. Curves 1, 2 and 3 correspond to methanol, ethanol and propanol, respectively.


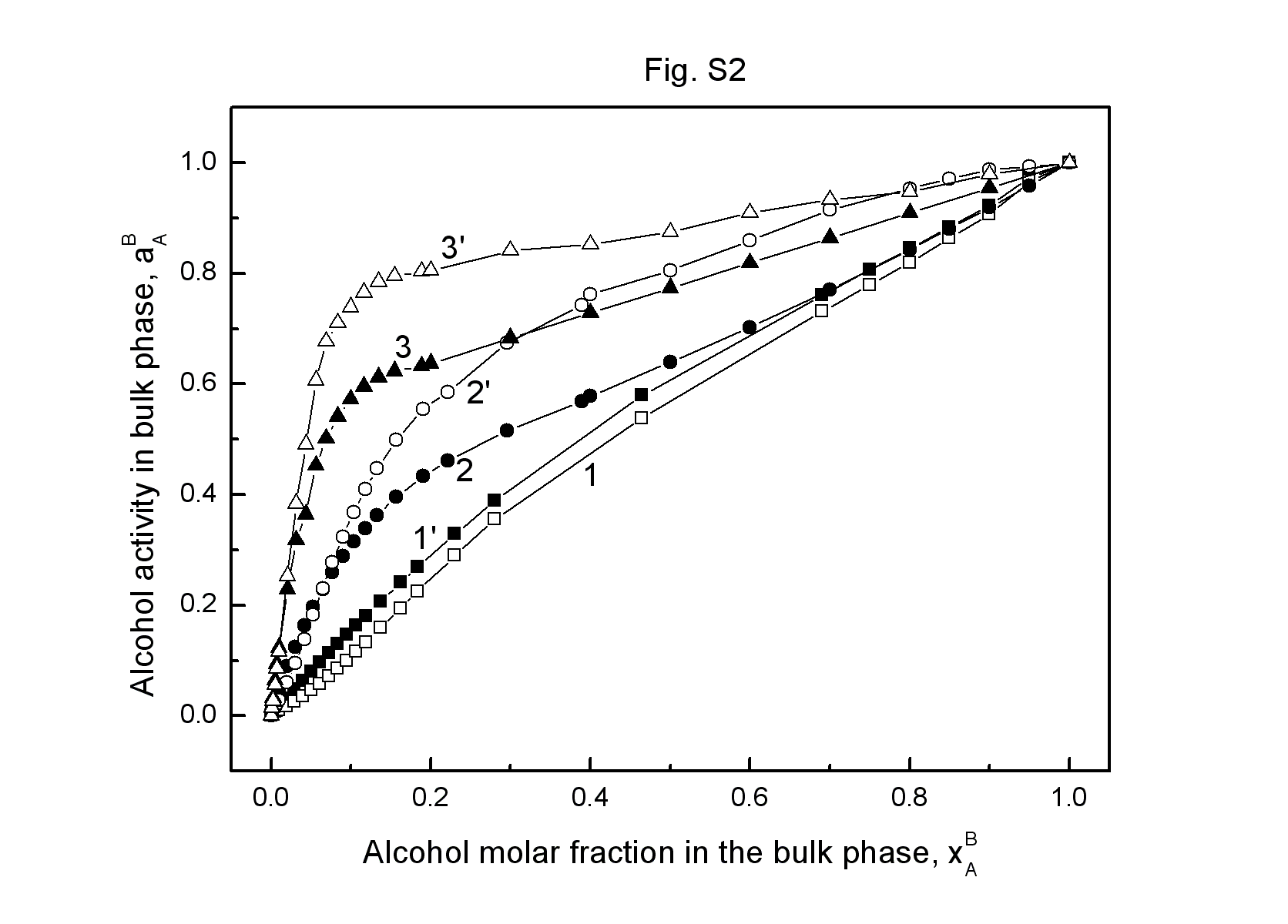


**Fig. S2** Dependence of the alcohol activity ($a_{A}^{B}$) on its molear fraction ($x_{A}^{B}$) in the bulk phase. Cures 1, 2 and 3 correspond to $a_{A}^{B}$ calculated from the Laar equation and curves 1’, 2’ and 3’ from Eq. 12 for methanol, ethanol and propanol, respectively.


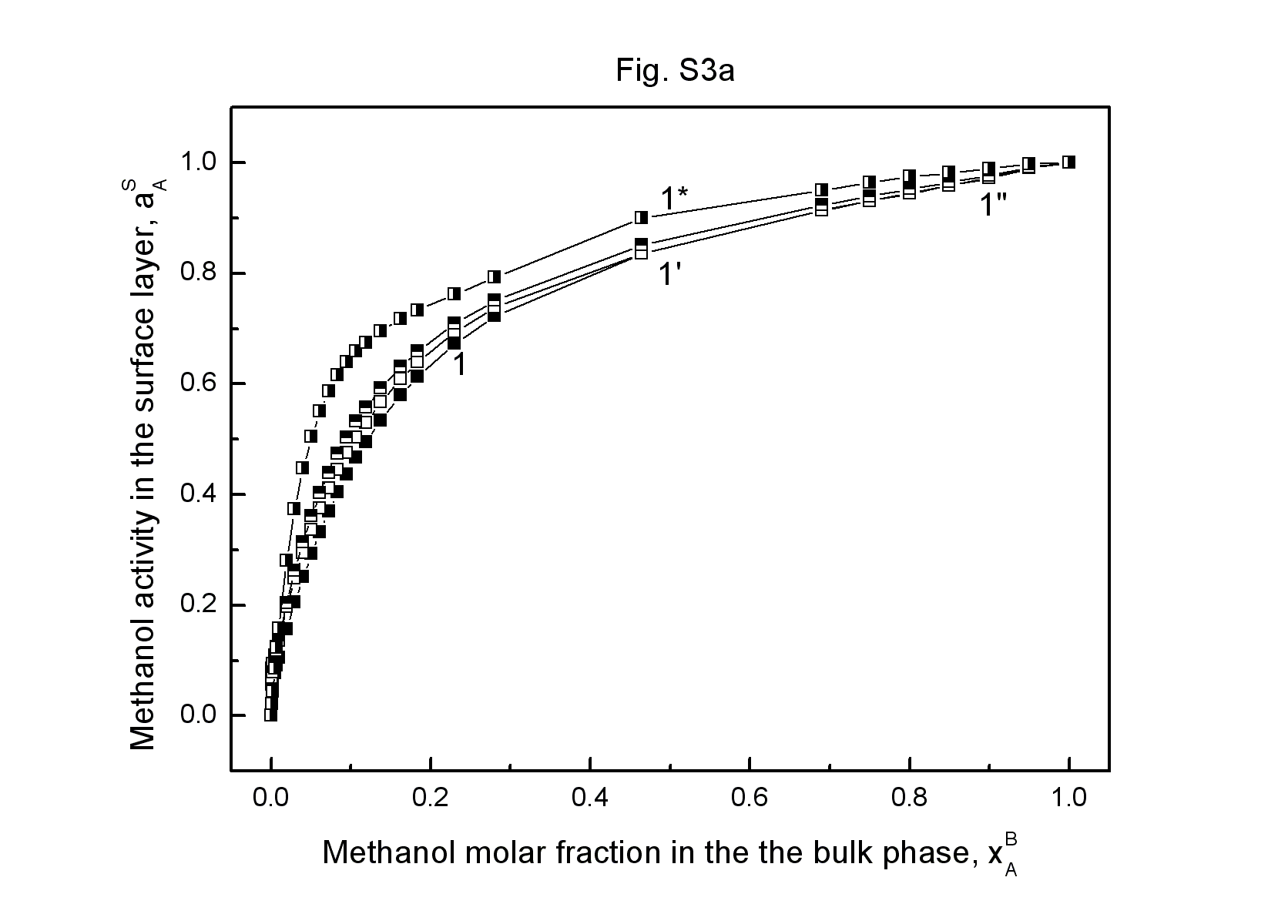


**Fig. S3a** Dependence of the methanol activity in the surface layer ($a_{A}^{S}$) on its mole fraction ($x_{A}^{B}$) in the bulk phase. Curves 1, 1’, 1” and 1* correspond to $a_{A}^{S}$ determined on the basis of Eq. 17 (curve 1), Eq. 11 (when $a_{A}^{B}$ was calculated from the Laar equation – curve 1’ and Eq. 12 – curve 1” ) and Eq. 12 (curve 1*), respectively


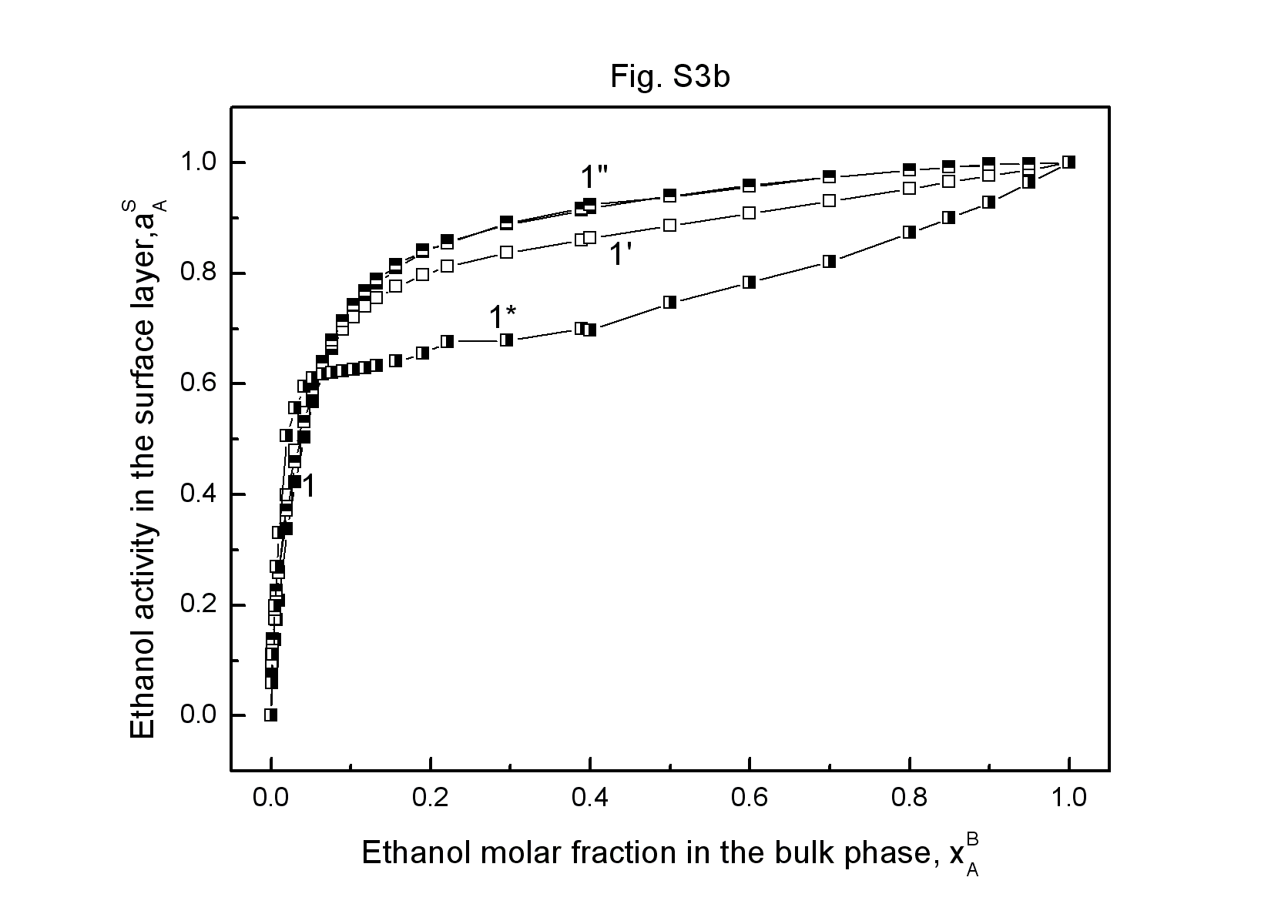


**Fig. S3b** Dependence of the ethanol activity in the surface layer ($a_{A}^{S}$) on its mole fraction ($x_{A}^{B}$) in the bulk phase. Curves 1, 1’, 1” and 1* correspond to $a_{A}^{S}$ determined on the basis of Eq. 17 (curve 1), Eq. 11 (when $a_{A}^{B}$ was calculated from the Laar equation – curve 1’ and Eq. 12 – curve 1” ) and Eq. 12 (curve 1*), respectively


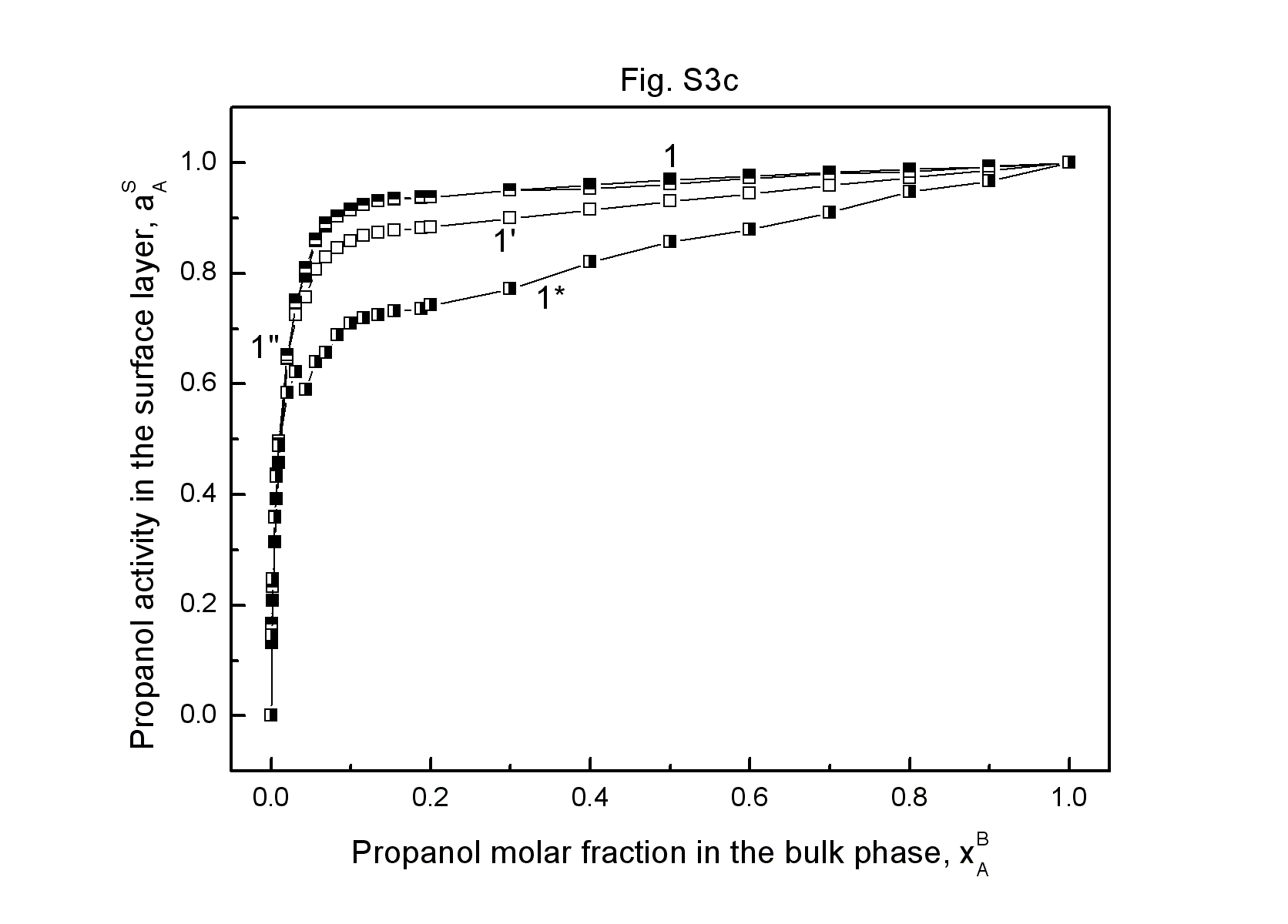


**Fig. S3c** Dependence of the propanol activity in the surface layer ($a_{A}^{S}$) on its mole fraction ($x_{A}^{B}$) in the bulk phase. Curves 1, 1’, 1” and 1* correspond to $a_{A}^{S}$ determined on the basis of Eq. 17 (curve 1), Eq. 11 (when $a_{A}^{B}$ was calculated from the Laar equation – curve 1’ and Eq. 12 – curve 1” ) and Eq. 12 (curve 1*), respectively


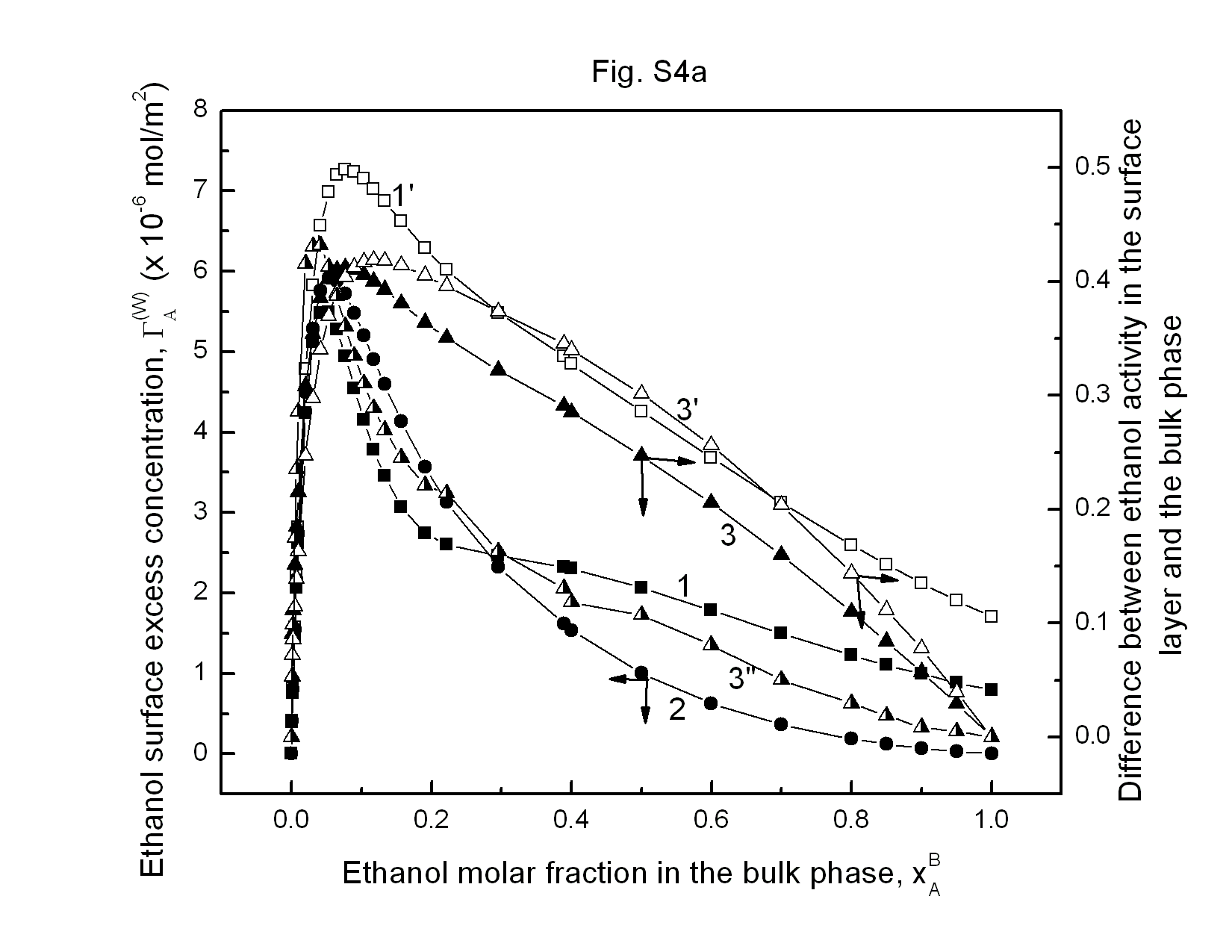


**Fig. S4a** A plot of the ethanol surface excess concentration ($\Gamma$) (curves 1–3) and differences between ethanol activity in surface layer at solution-air interface ($a_{A}^{S}$) and in the bulk phase ($a_{A}^{B}$) (curves 4–6) vs. ethanol mole fraction in the bulk phase ($x_{A}^{B}$). Curves 1 and 2 correspond to ethanol surface excess concentration calculated from Gibbs equation by using $x_{A}^{B}$and $a_{A}^{B}$, respectively, curve 3 to the ethanol surface excess concentration calculated from Eq. 18 and curves 4–6 correspond to $a_{A}^{S}$ − $a_{A}^{B}$ ($a_{A}^{S}$- determined on the basis of Eqs. 11, 17 and 12), respectively.


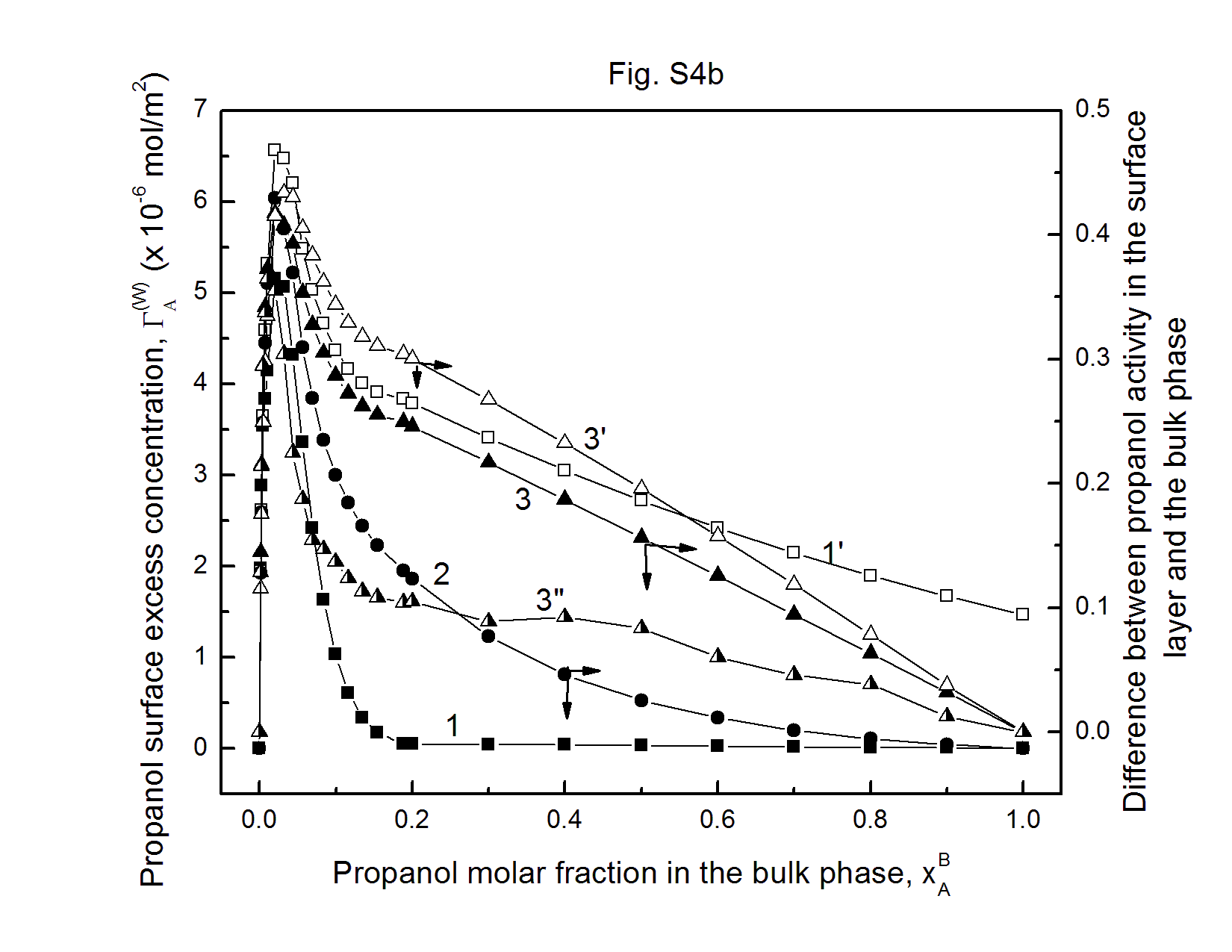


**Fig. S4b** A plot of the propanol surface excess concentration ($\Gamma$) (curves 1–3) and differences between propanol activity in surface layer at solution-air interface ($a_{A}^{S}$) and in the bulk phase ($a_{A}^{B}$) (curves 4–6) vs. propanol moer fraction in the bulk phase ($x_{A}^{B}$). Curves 1 and 2 correspond to propanol surface excess concentration calculated from Gibbs equation by using $x_{A}^{B}$and $a_{A}^{B}$, respectively, curve 3 to the propanol surface excess concentration calculated from Eq. 18 and curves 4–6 correspond to $a_{A}^{S}$ − $a_{A}^{B}$ ($a_{A}^{S}$- determined on the basis of Eqs. 11, 17 and 12), respectively


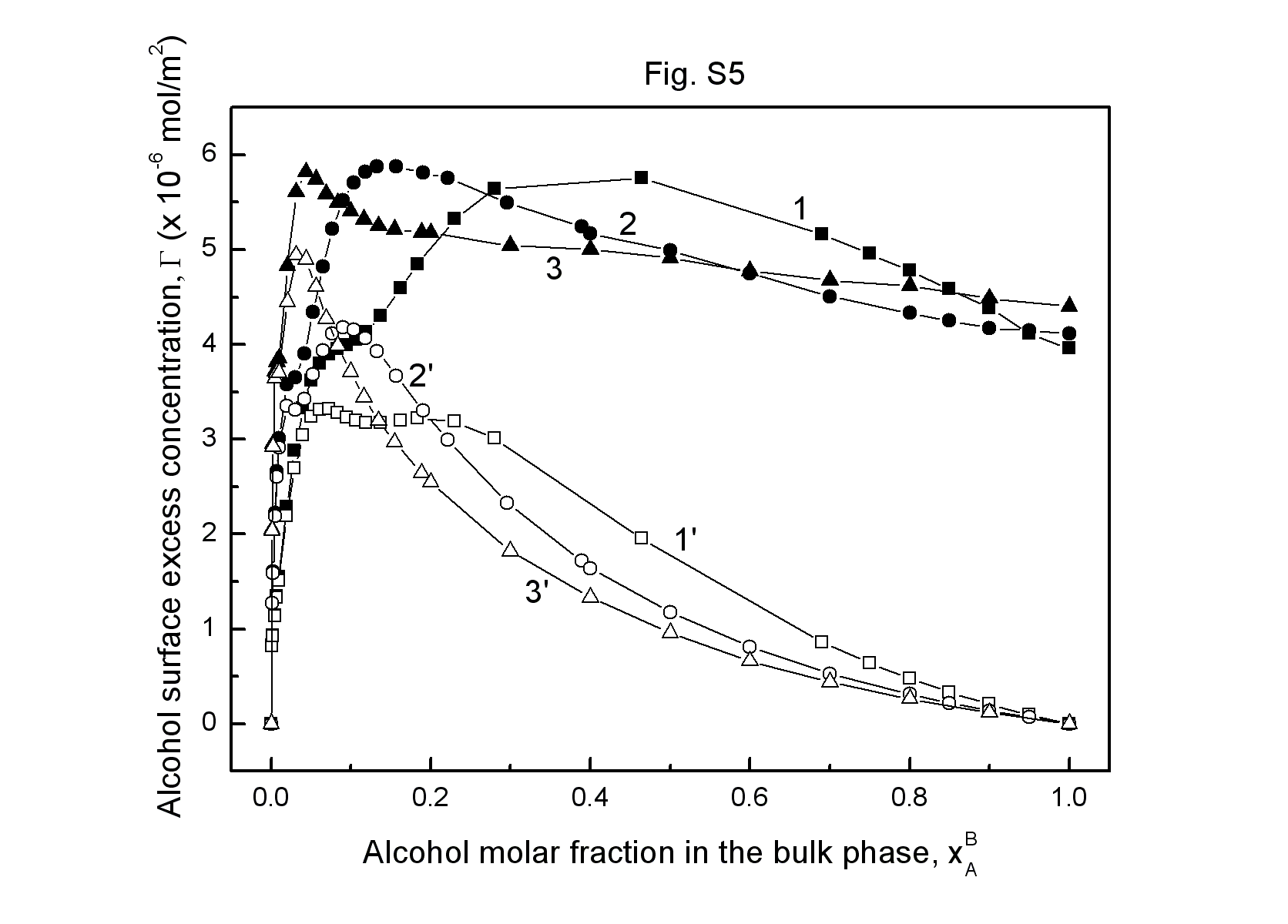


**Fig. S5** Dependence of the alcohol surface excess concentration (Γ) on its molar fraction in the bulk phase ($x_{A}^{B}$). Curves 1, 2 and 3 correspond to the methanol, ethanol and propanol surface excess concentration calculated from Gibbs equation (Eq. 7a) on the basis of alcohol activity in the bulk phase determined from Eq. 12 and curves 1’, 2’ and 3’ methanol, ethanol and propanol surface excess concentration calculated from Eq. 18, respectively


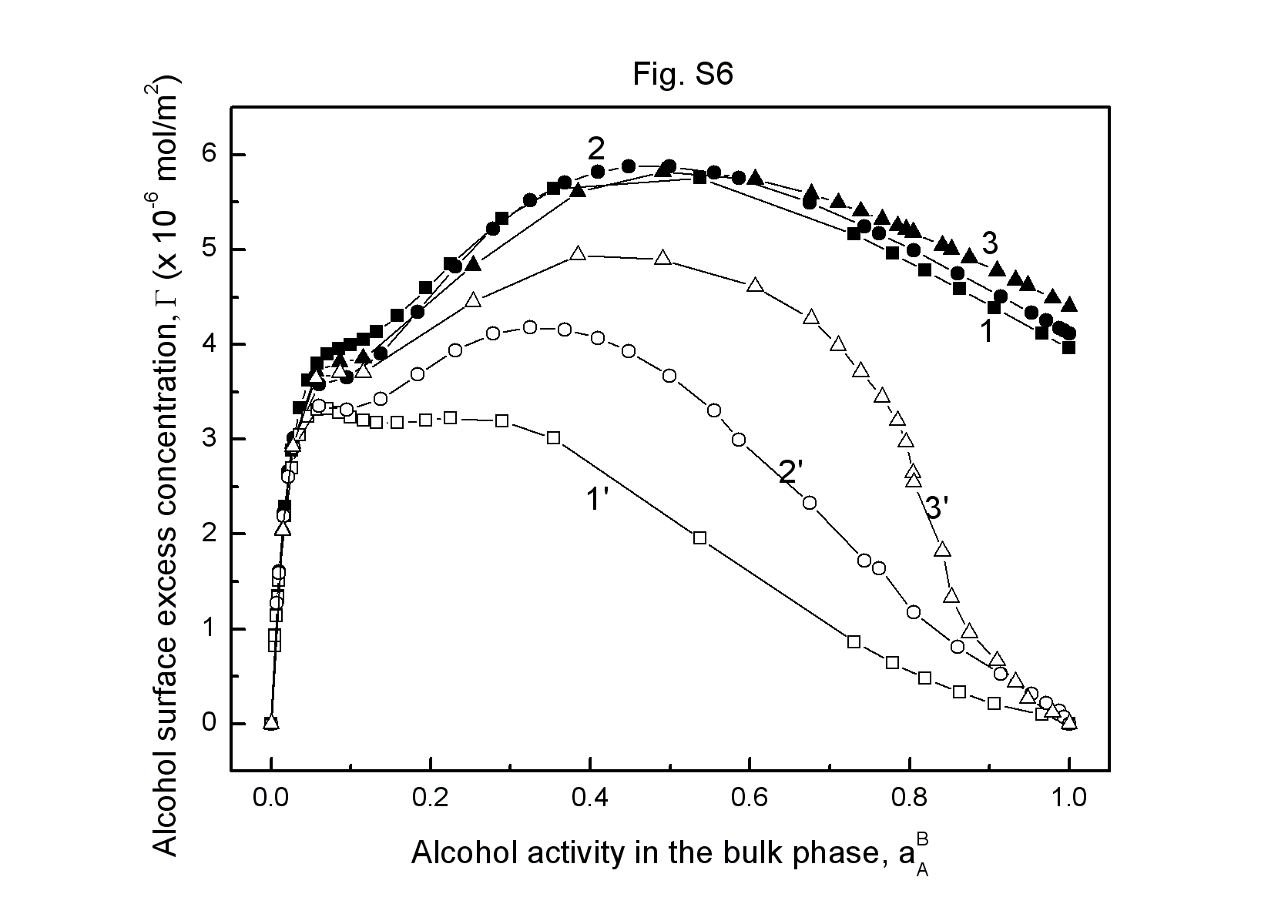


**Fig. S6** Dependence of the alcohol surface excess concentration (Γ) on its activity in the bulk phase ($a_{A}^{B}$) calculated from Eq. 12. Curves 1, 2 and 3 correspond to the methanol, ethanol and propanol surface excess concentration calculated from Gibbs equation (Eq. 7a) on the basis of alcohol activity in the bulk phase determined from Eq. 12 and curves 1’, 2’ and 3’ methanol, ethanol and propanol surface excess concentration calculated from Eq. 18, respectively


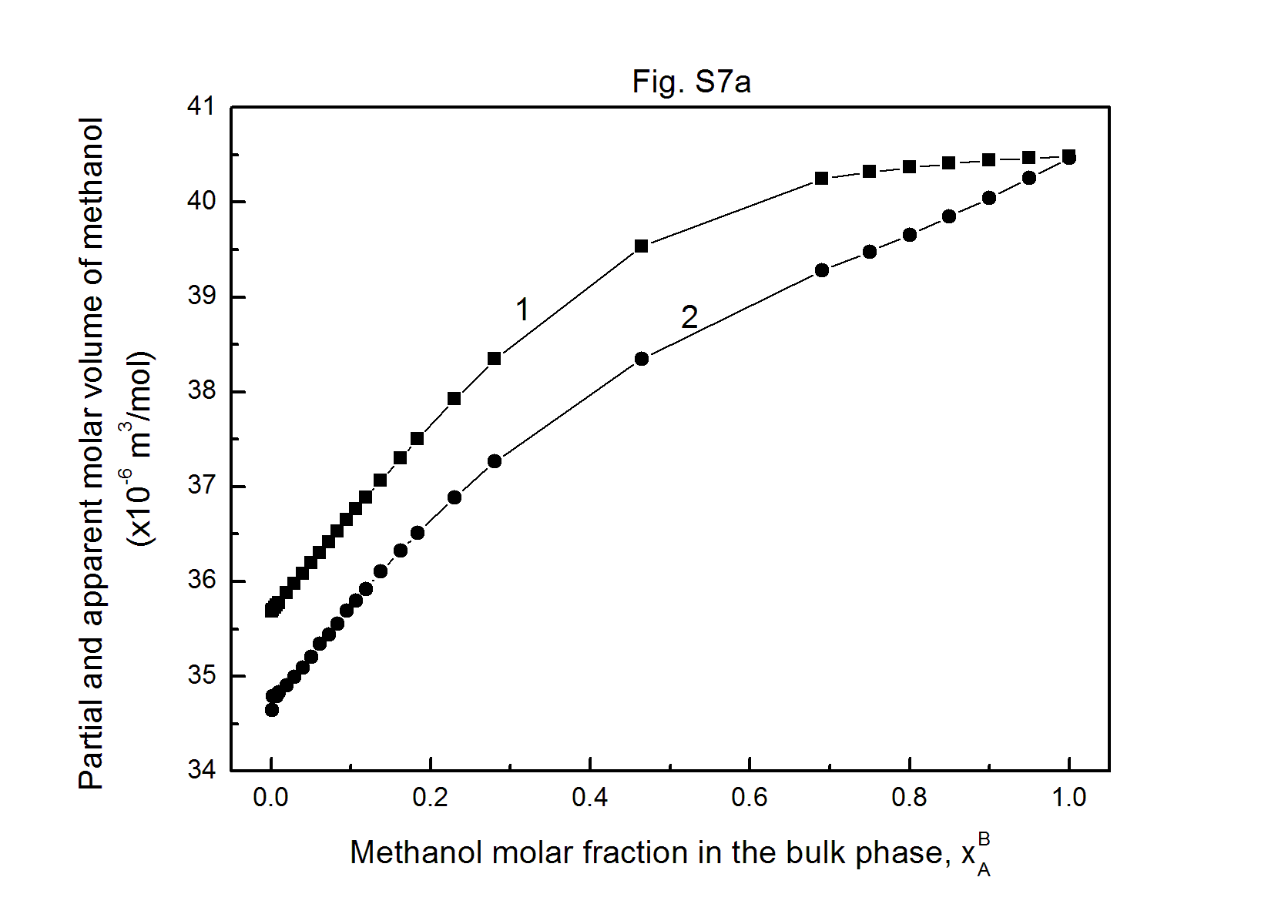


**Fig. S7a** A plot of partial ($\overline{V}_{M}$) and apparent ($\varphi_{V}$) methanol volume vs. mole fraction in the bulk phase ($x_{A}^{B}$). Curves 1 and 2 correspond to ($\overline{V}_{M}$) and $\varphi_{V}$, respectively


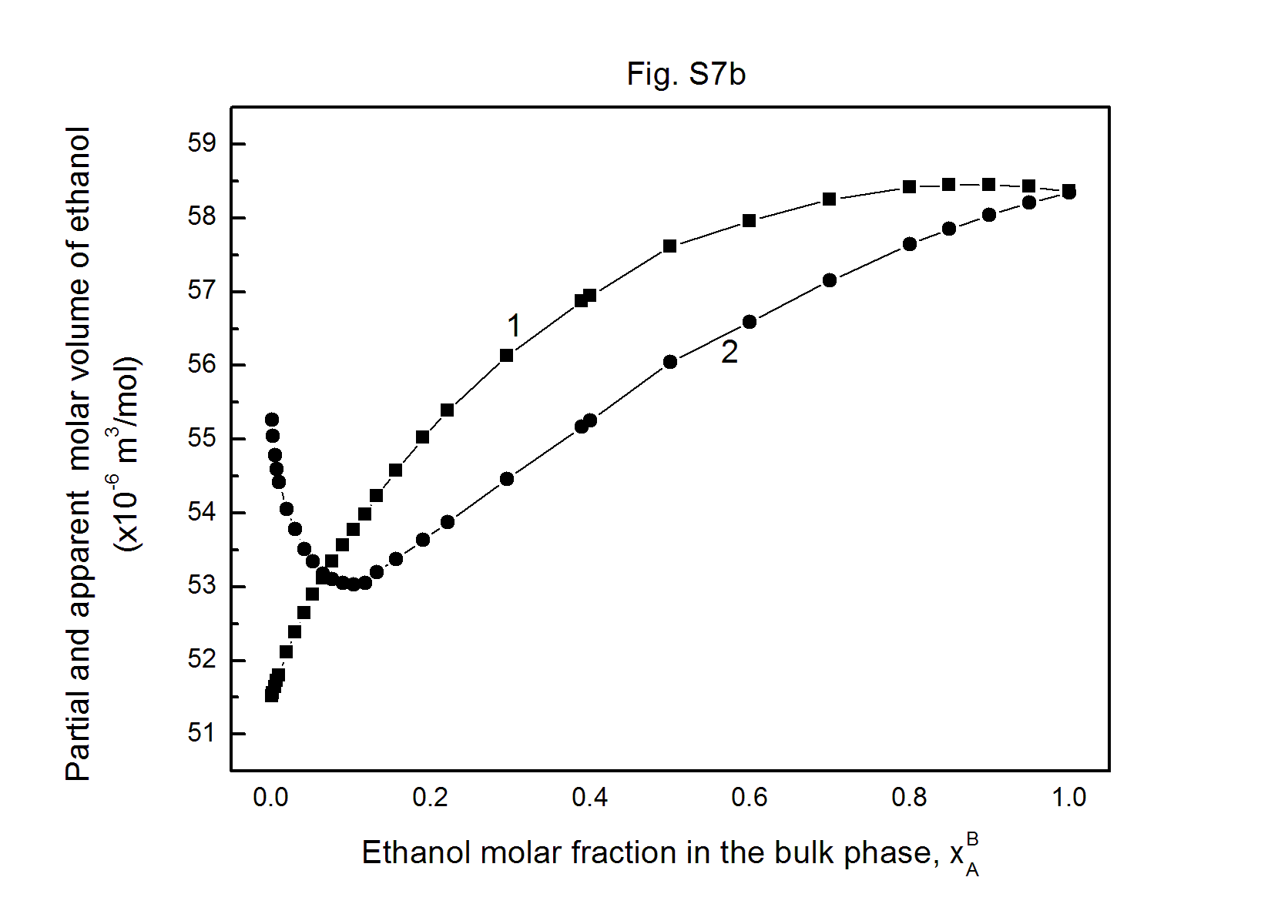


**Fig. S7b** A plot of partial ($\overline{V}_{M}$) and apparent ($\varphi_{V}$) ethanol volume vs. mole fraction in the bulk phase ($x_{A}^{B}$). Curves 1 and 2 correspond to ($\overline{V}_{M}$) and $\varphi_{V}$, respectively


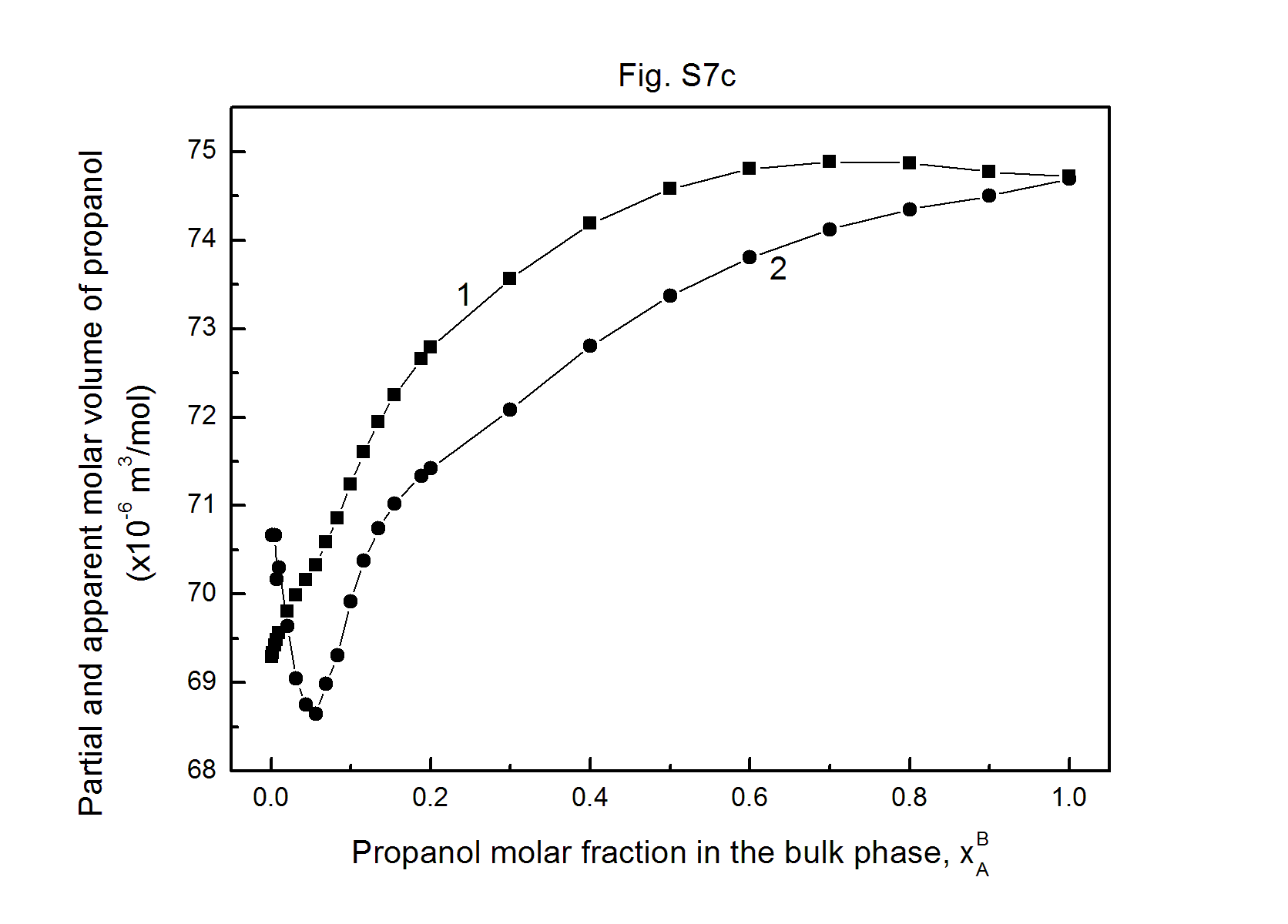


**Fig. S7c** A plot of partial ($\overline{V}_{M}$) and apparent ($\varphi_{V}$) propanol volume vs. mole fraction in the bulk phase ($x_{A}^{B}$). Curves 1 and 2 correspond to ($\overline{V}_{M}$) and $\varphi_{V}$, respectively.


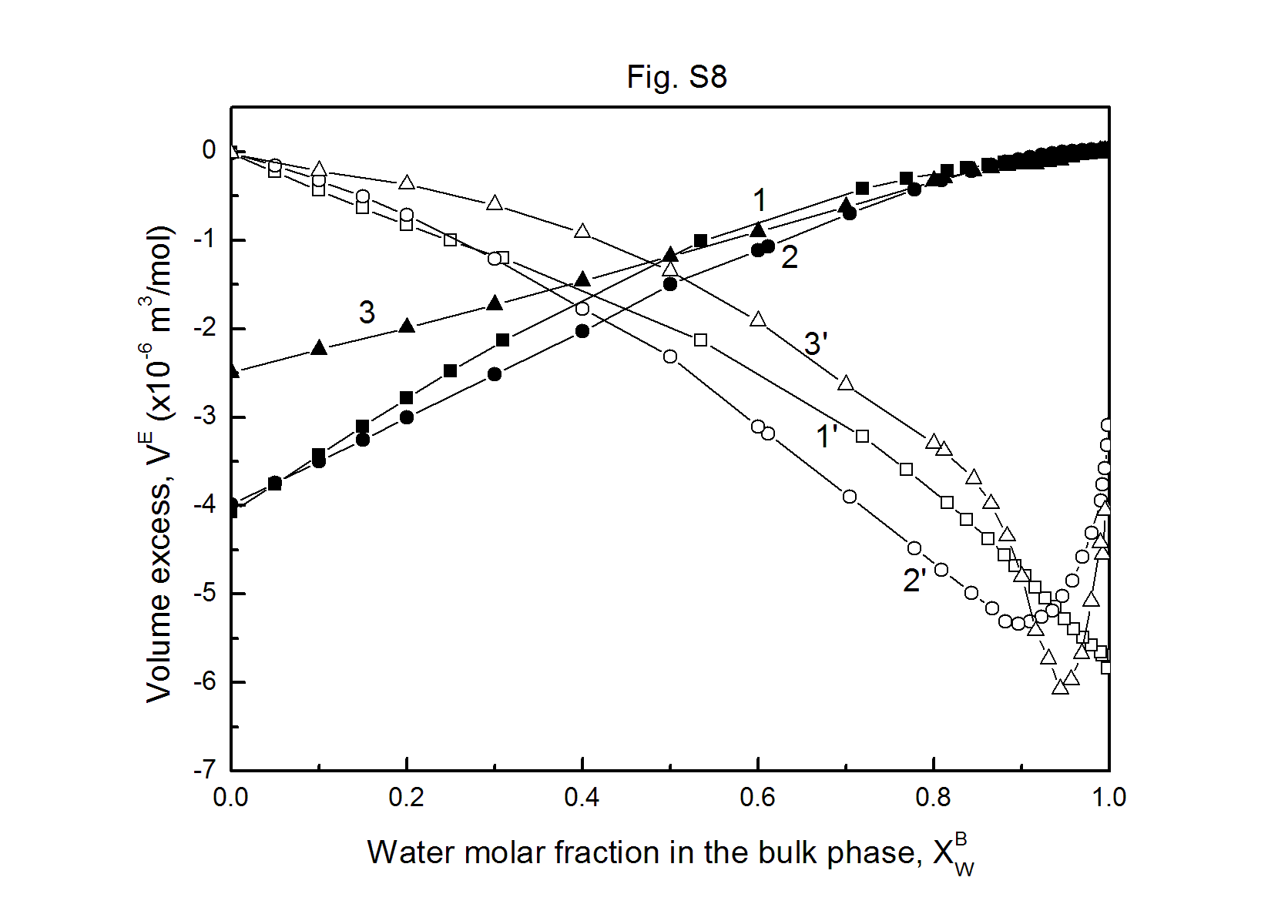


**Fig. S8** A plot of excess volume of water in methanol (curve 1), ethanol (curve 2) and propanol (curve 3), and methanol (curve 1’), ethanol (curve 2’) and propanol (curve 3’) in water vs. water mole fraction in aqueous solution of alcohol


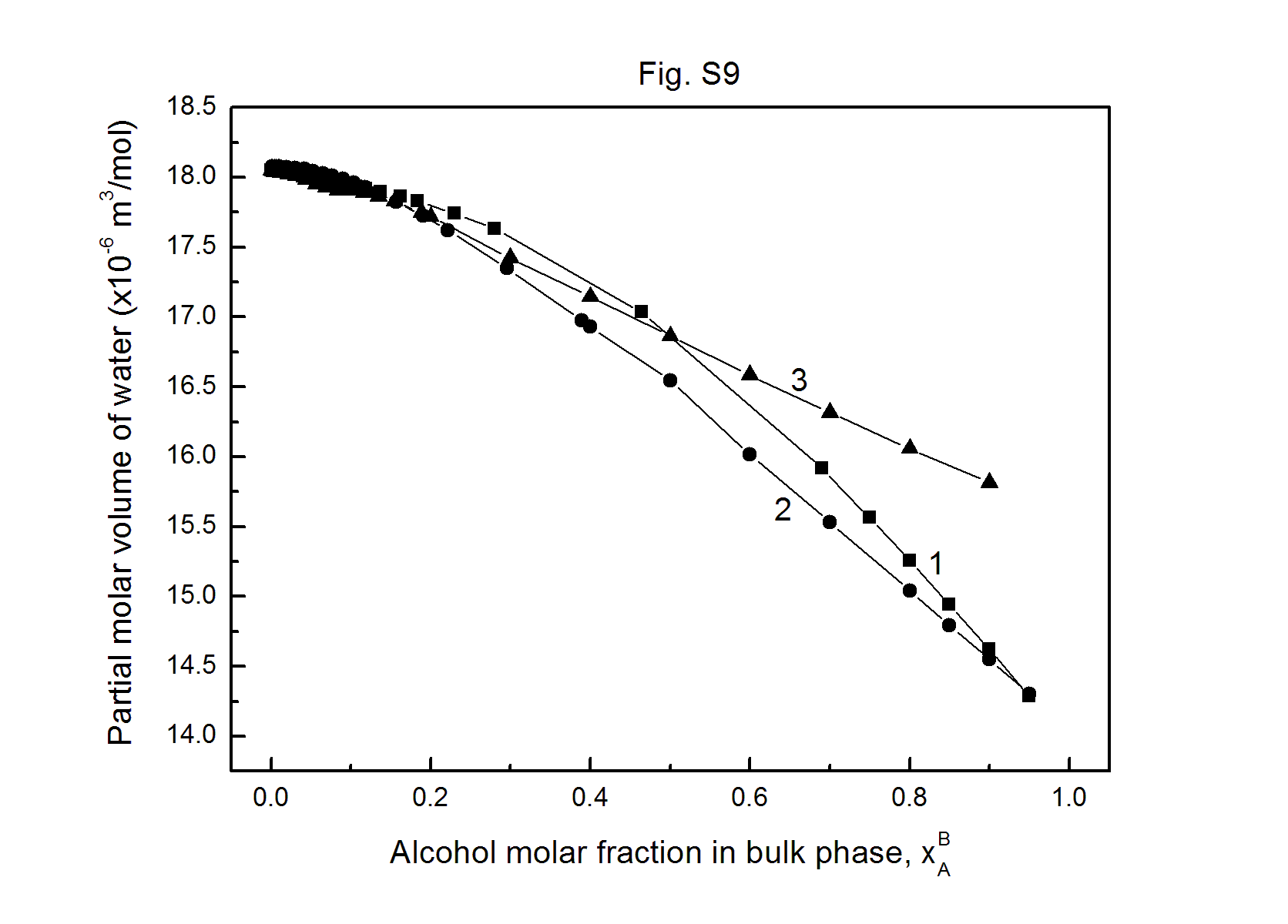


Fig. S9 Dependence of the partial molar volume ($\overline{V}_{M}$) of water on alcohol molar fraction in the bulk phase ($x_{A}^{B}$). Curves 1, 2 and 3 correspond to the water partial volume in methanol, ethanol and propanol, respectively.
